# Gene‐Metabolite Network Linked to Inhibited Bioenergetics in Association With Spaceflight‐Induced Loss of Male Mouse Quadriceps Muscle

**DOI:** 10.1002/jbmr.4102

**Published:** 2020-07-30

**Authors:** Nabarun Chakraborty, David L Waning, Aarti Gautam, Allison Hoke, Bintu Sowe, Dana Youssef, Stephan Butler, Michael Savaglio, Paul J Childress, Raina Kumar, Candace Moyler, George Dimitrov, Melissa A Kacena, Rasha Hammamieh

**Affiliations:** ^1^ The Geneva Foundation Walter Reed Army Institute of Research Silver Spring MD USA; ^2^ Medical Readiness Systems Biology Walter Reed Army Institute of Research Silver Spring MD USA; ^3^ Penn State College of Medicine Hershey PA USA; ^4^ Oak Ridge Institute for Science and Education (ORISE) Walter Reed Army Institute of Research Silver Spring MD USA; ^5^ Department of Orthopaedic Surgery Indiana University School of Medicine Indianapolis IN USA; ^6^ Richard L. Roudebush VA Medical Center Indianapolis IN USA

**Keywords:** ANIMAL MODEL, METABOLISM, SKELETAL MUSCLE, SYSTEMS BIOLOGY, TISSUE SIGNALING

## Abstract

Prolonged residence of mice in spaceflight is a scientifically robust and ethically ratified model of muscle atrophy caused by continued unloading. Under the Rodent Research Program of the National Aeronautics and Space Administration (NASA), we assayed the large‐scale mRNA and metabolomic perturbations in the quadriceps of C57BL/6j male mice that lived in spaceflight (FLT) or on the ground (control or CTR) for approximately 4 weeks. The wet weights of the quadriceps were significantly reduced in FLT mice. Next‐generation sequencing and untargeted mass spectroscopic assays interrogated the gene‐metabolite landscape of the quadriceps. A majority of top‐ranked differentially suppressed genes in FLT encoded proteins from the myosin or troponin families, suggesting sarcomere alterations in space. Significantly enriched gene‐metabolite networks were found linked to sarcomeric integrity, immune fitness, and oxidative stress response; all inhibited in space as per in silico prediction. A significant loss of mitochondrial DNA copy numbers in FLT mice underlined the energy deprivation associated with spaceflight‐induced stress. This hypothesis was reinforced by the transcriptomic sequencing–metabolomics integrative analysis that showed inhibited networks related to protein, lipid, and carbohydrate metabolism, and adenosine triphosphate (ATP) synthesis and hydrolysis. Finally, we discovered important upstream regulators, which could be targeted for next‐generation therapeutic intervention for chronic disuse of the musculoskeletal system. © 2020 The Authors. *Journal of Bone and Mineral Research* published by American Society for Bone and Mineral Research.

## Introduction

Extended periods of skeletal muscle disuse that is prevalent in many populations such as the elderly,^(^ ^1^ ^)^ the injured,^(^ ^2^ ^)^ and astronauts^(^ ^3^ ^)^ lead to significant losses of muscle mass, morphology, and strength. In bed rest studies simulating unloading condition, losses of 6% to 11% of quadriceps mass and power have been reported in as little as 3 weeks.^(^ ^4^ ^)^ For spaceflight, both long‐term (up to 6 months) and short‐term (1 to 2 weeks) space missions reported losses in quadriceps volume of up to 15%, with similar numbers reported for loss of strength.^(^ ^5^, ^6^ ^)^ In support, significant atrophy of rodent quadriceps was observed after a 30‐day‐long space mission^(^ ^7^ ^)^ and in 14‐day‐long and 21‐day‐long hindlimb unloading studies (to model disuse).^(^ ^8^ ^)^ A common theme across these types of studies is that the thigh muscles which include quadriceps are typically affected by unloading. The dose‐dependent effect of unloading is somewhat alleviate in astronauts, because they are required to follow various exercise protocols on any space mission in which they participate.^(^ ^9^ ^)^ This suggests that the atrophy in space could be much greater than that observed in bed rest if the astronauts were able to remain inactive for extended periods in space. Clinical studies using astronauts are further limited due to the small sample size.

Rodent research at the International Space Station (ISS) is a comparatively new undertaking. The National Aeronautics and Space Administration (NASA)’s rodent spaceflight hardware is capable of housing only 40 mice at a time with certain provisions as necessary to conduct such research in space. Taking full advantages of sample size and other means offered at the ISS, our project, named as Rodent Research 4 (https://www.nasa.gov/ames/research/space‐biosciences/rodent‐research‐4‐spacex‐10), housed male mice in NASA’s hardware at the ISS for the first time. A ground‐based control study (CTR) used the same rodent habitat conditions, mimicking all the procedures performed at ISS, offset by 5 days. Wet weights of various muscles underscored the detrimental effects of spaceflight‐induced stress on muscle volume. Quadriceps muscles, previously identified for displaying significant impacts from spaceflight and its simulating environments,^(^ ^3^, ^4^, ^5^, ^6^ ^)^ were selected to divulge the molecular underpinnings of the muscle loss. A deep gene sequencing readout suggested mitochondrial dysfunction; hence, the global metabolomics of quadriceps was conducted to examine the bioenergetics profile. Functional analysis trained toward muscular atrophy found comprehensive inhibition of key molecular networks essential for normal muscular functions. In addition, we determined a set of quadriceps‐specific upstream regulators, which could have significant clinical interest, because a large number of genes and their biofunctions can be systematically controlled via targeting a single upstream regulator.^(^ ^10^ ^)^

## Materials and Methods

### Ethics statement

All animal experiments were approved by the ISS and Kennedy Space Center (KSC) Institutional Animal Care and Use Committees (IACUCs), and were performed in facilities accredited by the Association for Assessment and Accreditation of Laboratory Animal Care International (AAALAC). This research complied with the Animal Welfare Act and implementing Animal Welfare Regulations, the Public Health Service Policy on Humane Care and Use of Laboratory Animal.

### Animal handling

Details of the animal handling protocols are presented in the online Supplementary Data. Briefly, 9‐week‐old, male C57BL/6j mice were used for this study. Cages of 10 mice were randomly selected to be housed on the ISS (spaceflight [FLT]) or at KSC (CTR). The ground control study at KSC began with a 5‐day offset. Both FLT and CTR mice were housed under similar caging conditions including 12‐hour light/dark cycle and were provided with the same diet/water *ad libitum*. Mice were humanely euthanized 24 to 28 days post‐launch when the mice were ~13weeks of age. Five of 10 mice/group were reserved for imaging studies.^(^ ^11^ ^)^ For the remaining five mice, the carcasses were snap‐frozen at –80°C. FLT samples were returned to Earth; and all samples were processed at the same time in the same laboratory. Therefore, a maximum of five mice/group were available for these studies. Four randomly selected FLT and CTR carcasses were used for omics analyses. The muscle tissues were isolated and frozen at –80°C for further analysis.

Upon retrieving the right leg quadriceps, extensor digitorum longus (EDL), and soleus muscle tissue from the freezer, their wet weights were recorded. Next, we proceeded with the quadriceps tissue for multiomics assay. The quadriceps was cryogenically homogenized using a Cryomill (Haan, Germany). An aliquot of the homogenate was allotted to extract DNA and RNA using the standard TRIzol (Invitrogen, Thermo Fisher Scientific, Wilmington, MA, USA) method. The copy number variation of mitochondrial DNA was measured using NovaQUANT (Novagen, Madison, WI, USA). For large‐scale mRNA sequencing, 2 × 150 cycles of paired‐end reads were generated in HiSeq4000 platform (Illumina, Inc., San Diego, CA, USA) following TruSeq stranded mRNA protocol. The reads were trimmed, normalized with the weighted trimmed mean of M‐values method and aligned to *Mus musculus* whole genome assembly (mm10; https://genome.ucsc.edu/cgi-bin/hgGateway?db=mm10). The data file is uploaded at the Sequence Read Archive (SRA; https://www.ncbi.nlm.nih.gov/sra/); submission ID: SUB6956586.

A second aliquot of quadriceps homogenate was used for global metabolomics analysis using mass spectrometry performed on a Quadrupole Time‐of‐Flight (Q‐TOF) Premier mass spectrometer (Waters Corporation, Milford, MA, USA). The differentially expressed mass spectroscopy peaks were annotated using CEU Mass Mediator database (http://ceumass.eps.uspceu.es/) and the molecules were screened based on the following guideline: (i) parts per million (ppm) error <1; (ii) chemical formula comprised of the adducts: +H, −H, +Na, +K, +NH4, and −Cl; (iii) annotate by Human Metabolome Database (HMDB; https://hmdb.ca/); and (iv) chemical type of one of the following categories: (i) endogenous mammalian, (ii) drugs, (iii) toxicant, and (iv) reagents. The third aliquot was used for estimating the load of myosin protein using and ELISA kit from Abbexa (Cambridge, UK).

The statistical analysis was performed using R scripts (R Foundation for Statistical Computing, Vienna, Austria; https://www.r‐project.org/) and GeneSping (Agilent Technologies, Santa Clara, CA, USA). The differentially expressed transcripts and metabolites were selected via moderate *t* test *p* < .05. mRNA and metabolite co‐enriched networks were mined using Ingenuity Pathway Analysis (IPA) v01‐13 (QIAGEN, Inc., Hilden, Germany) and ImPala v12.^(^ ^12^, ^13^ ^)^ The networks were selected based on two criteria: (i) hypergeometric *p* < .05; and (ii) number of molecules (gene and/or metabolite) >5. *Z*‐score was used to define the regulations of networks; *Z*‐scores >1.5 and < −1.5 were considered activated or inhibited networks, respectively. To curate the upstream regulators of interest, we applied the following criteria: (i) the upstream regulator should either be differentially expressed gene (DEG) or differentially expressed metabolite (DEM); (ii) *Z*‐score > |1.5|; and (iii) hypergeometric *p* < .05.

## Results

**Fig 1:**
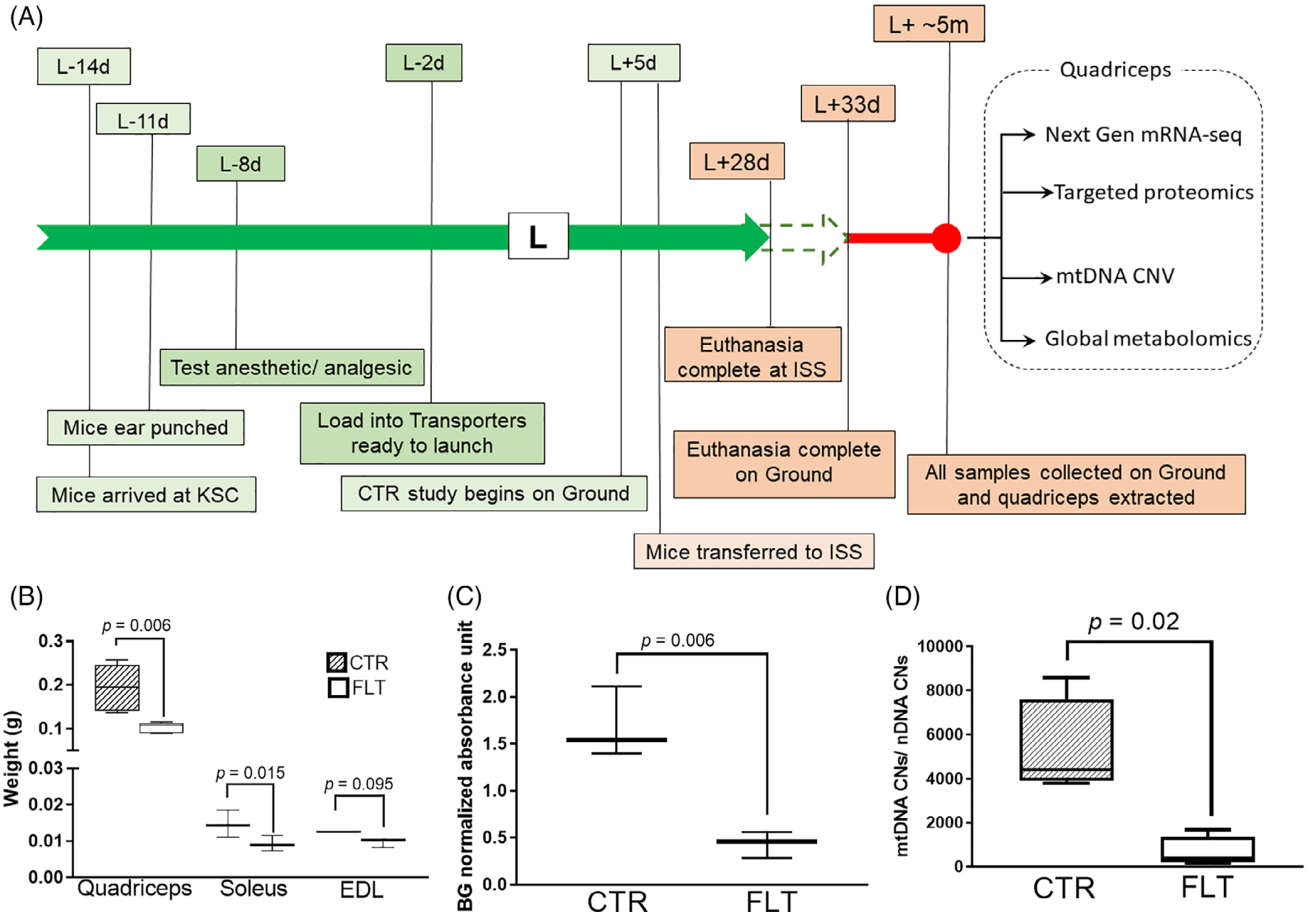
(*A*) The timeline of Rodent Research 4 (RR4). The timeline depicts how the research was planned around the launch (L) of space shuttle SpaceX‐10. The types of assays reported in this article are listed at the end. Timeline is not to scale. (*B*) The wet mass of quadriceps, soleus, and EDL. The bar and whisker plot represents the median weight values with the 10 to 90 percentile. The hashed and white boxes represents FLT and CTR mice cohorts, respectively. The significance level of changes was calculated using Student’s *t* test. (*C*) The regulation of myosin protein in FLT versus CTR mice. The bar and whisker plot presents the median values with 10 to 90 percentile. The reduction of OD_450_ in FLT (white bar) in comparison to the CTR (hashed bar) was significant. (*D*) mtDNA CNV in FLT versus CTR mice. The *y* axis represents the ratio of CNs of mtDNA and nDNA. The bar and whisker plot presents the median values with 10 to 90 percentile. The significance level of changes was calculated using Student’s *t* test. BG = background; CN = copy number; CTR = control group on the ground; d = day; EDL = extensor digitorum longus; FLT = spaceflight; ISS = International Space Station; KSC = Kennedy Space Center; L = launch; m = months; mtDNA CNV = mitochondrial DNA copy number variation; nDNA = nuclear DNA; OD_450_ = optical density at 450 nm; w = weeks.

Cages of 10 male C57BL/6j mice at ~9 weeks of age were randomly assigned into FLT or CTR groups. FLT mice were launched in space and stayed there for nearly 4 weeks. Both groups were humanely euthanized in identical manners in spaceflight or on the ground (asynchronous 5 days) (Fig. 1*A*). All the frozen carcasses were collected on Earth and the tissues were extracted within 20 min after undergoing minimum freeze‐thaw cycle. Omics assays were conducted on four randomly selected mice per group.

Wet weights of the right leg quadriceps, soleus, and EDL muscles were decreased by 50% (*p* = .006), 36% (*p* = .095), and 23% (*p* = .015), respectively, in the FLT mice compared to CTR mice (Fig. 1*B*). There was a significant (*p* < .01, Student’s *t* test) reduction in the myosin load of quadriceps from FLT mice as compared to CTR mice, as evidenced by a decline of nearly 30% in the ELISA optical density measurement (Fig. 1*C*). The mitochondrial DNA copy number variation (mtDNA CNV) assay found a significant sevenfold decrease of mtDNA CNs in FLT quadriceps in comparison to CTR quadriceps (*p* = .020, Student’s *t* test) (Fig. 1*D*).

Quality controlled mRNA reads of quadriceps tissue (Fig. S1) were successfully mapped onto 10,874 genes (~54% of whole mouse genes) with counts per million >0.5 in all eight samples (*n* = 4/group × FLT and CTR). The detail of the data quality is narrated in the online Supplementary Data. There were 776 DEGs between FLT and CTR quadriceps, which included 378 upregulated and 398 downregulated genes. False discovery rate (FDR) correction yielded 19 genes that met the cutoff 0.1 (Table 1), whereas 14 of these 19 genes met the FDR cutoff of 0.05.

**Table 1:** The 19 Quadriceps‐Specific Genes, Differentially Expressed Between the Spaceflight (FLT) and Ground Control (CTR) Mice, Meeting FDR Cutoff 0.1

Gene symbol	Log_2_(fold change)	*p*	FDR	Entrez gene name	Location of encoded protein	Type of encoded protein
TNNT1	−3.63	1.72E‐08	1.87E‐04	Troponin T1, slow skeletal type	Cytoplasm	Other
MYH7	−5.23	1.25E‐07	6.79E‐04	Myosin heavy chain 7	Cytoplasm	Enzyme
TNNC1	−3.92	3.27E‐07	8.90E‐04	Troponin C1, slow skeletal and cardiac type	Cytoplasm	Other
TNNI1	−4.48	2.60E‐07	8.90E‐04	Troponin I1, slow skeletal type	Cytoplasm	Other
MYL2	−3.34	1.66E‐06	2.58E‐03	Myosin light chain 2	Cytoplasm	Other
MYLK4	−1.46	1.48E‐06	2.58E‐03	Myosin light chain kinase family member 4	Cytoplasm	Kinase
PTPN3	−1.77	1.63E‐06	2.58E‐03	Protein tyrosine phosphatase, non‐receptor type 3	Cytoplasm	Phosphatase
TPPP3	1.34	2.12E‐05	2.88E‐02	Tubulin polymerization promoting protein family member 3	Cytoplasm	Other
PER3	−1.53	2.71E‐05	3.28E‐02	Period circadian regulator 3	Nucleus	Other
AVIL	1.26	4.12E‐05	3.78E‐02	Advillin	Plasma membrane	Other
GOLGA7B	1.45	3.91E‐05	3.78E‐02	Golgin A7 family member B	Other	Other
LRRC30	1.02	4.52E‐05	3.78E‐02	Leucine rich repeat containing 30	Other	Other
MYOZ2	−2.24	4.40E‐05	3.78E‐02	Myozenin 2	Other	Other
4832428D23Rik	1.90	5.57E‐05	4.33E‐02	NA	NA	NA
MYH2	−1.47	7.76E‐05	5.62E‐02	Myosin heavy chain 2	Cytoplasm	Enzyme
TRIOBP	0.87	1.12E‐04	7.61E‐02	TRIO and F‐actin binding protein	Nucleus	Other
PER2	−2.43	1.31E‐04	8.36E‐02	Period circadian regulator 2	Nucleus	Transcription regulator
PFN2	−0.85	1.42E‐04	8.36E‐02	Profilin 2	Cytoplasm	Enzyme
DCAF4	0.88	1.68E‐04	9.60E‐02	DDB1 and CUL4 associated factor 4	Nucleus	Other

We report the gene symbol, gene name, FDR, and corresponding *p* values. The location and type of the protein encoded by the gene is reported as well. There are four genes (ie, MYH7, MYL2, MYLK4, and MYH2) in this list that encode proteins related to myosin family. Similarly, there are three genes (ie, TNNT1, TNNC1, and TNN1) in this list that encode proteins related to the troponin family. These are the two most popular protein families in this list. NA = not applicable.

Mass spectrometry analysis of quadriceps tissues identified 740 DEMs between FLT and CTR, which included 440 downregulated and 300 upregulated metabolites. None of these metabolites met FDR correction.

Biological network analysis was carried out using those DEGs and which met a moderate *t* test *p* < .05. There were 23 significantly enriched (Fisher’s hypergeometric *t* test *p* < .05) and significantly regulated (*Z*‐score > |1.5|) canonical networks (Table S1). The eukaryotic initiation factor 2 (eIF2) signaling networks emerged as the most significantly activated network (*Z*‐score > 1.5) in FLT mice compared to CTR quadriceps. In addition, there were 21 canonical networks significantly inhibited in FLT mice (*Z*‐score < −1.5). Integrin signaling and nuclear factor erythroid 2–related factor 2 (Nrf2)‐mediated oxidative stress response signaling networks were the two most inhibited canonical networks, which were enriched by 19 and 23 DEGs, respectively.

**Fig 2:**
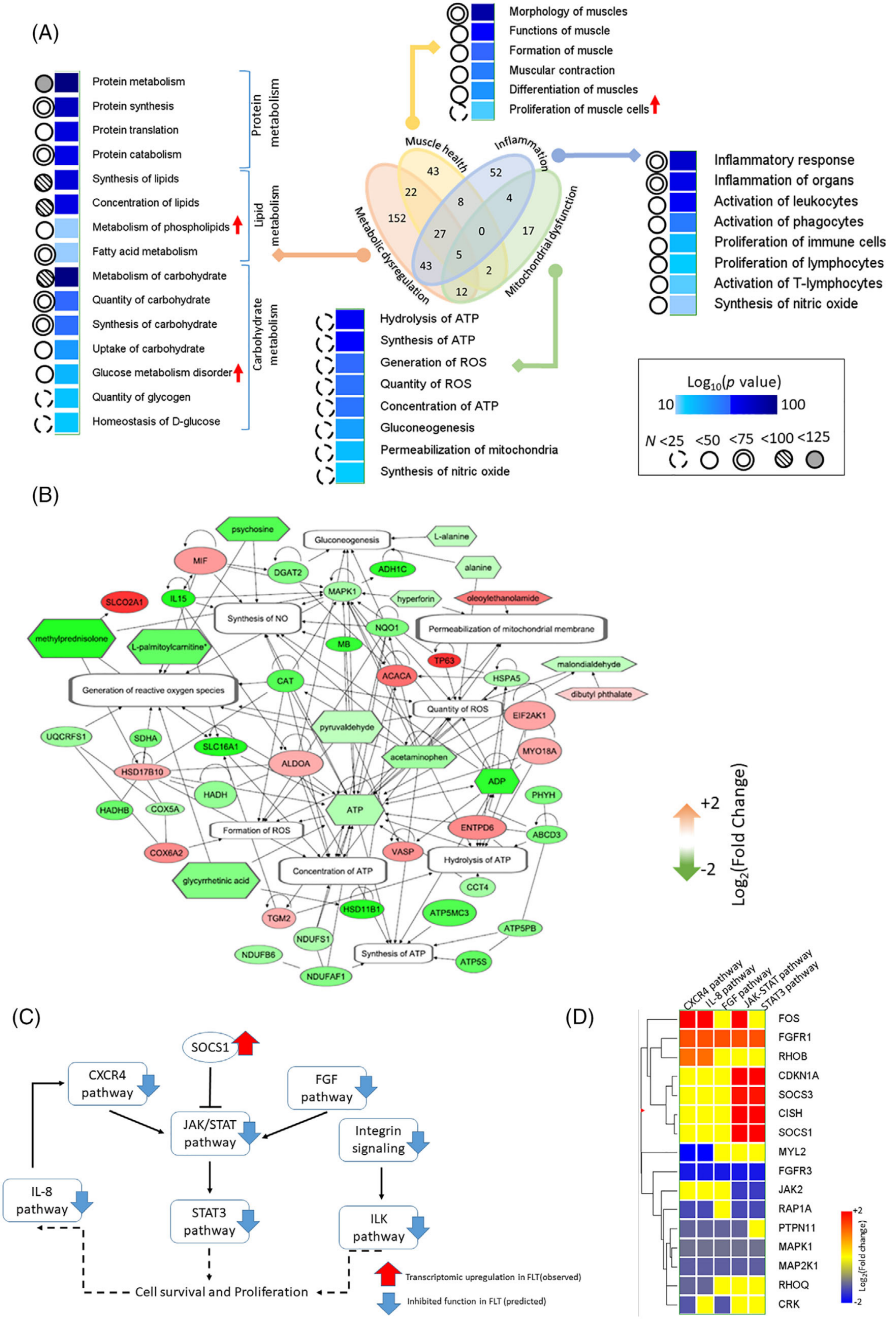
(*A*) Four noncanonical networks of interest, namely metabolic dysfunction, muscle health, inflammation, and mitochondrial dysregulation. The metabolic dysfunction includes dysfunctions in metabolism of carbohydrate, lipid, and protein, respectively. The Venn diagram at the center suggests the association among these four noncanonical functions, whereas the numbers inside the diagram represent overlapping DEGs and DEMs across the networks. The subnetworks under each noncanonical network are listed next to the color‐coded boxes representing their significance of enrichment calculated by hypergeometric *t* test. The color code of the range of log_10_(*p* values) is shown at the left bottom corner; two extreme points, namely the dark blue and light blue, represented by log_10_(*p* values) = 10 and 100, respectively. The numbers of molecules (DEGs + DECs) enriching each network are represented by different circles next to the columns of log_10_(*p* values). Circles bordered by broken line, solid line, double line, solid line + filled with hashed lines, and solid line + filled solid color represent the sample sizes <25, 50, 75, 100, and 125, respectively. The regulation status (activated versus inhibited) of the networks were computed by MAP algorithm. Only the activated networks were marked by upward red arrows; the remaining unmarked networks were inhibited as per MAP algorithm. (*B*) Bioenergetics networks inhibited in spaceflight. The oval, hexagonal, and rectangular shaped nodes represent gene, metabolite, and biofunction, respectively. Red color means upregulated and green means downregulated in spaceflight compared to controls. All of the biofunctions noted inside the clear rectangles were inhibited in spaceflight as predicted by the MAP algorithm supplied by IPA. The arrow‐headed and open‐ended edges represent the regulative and associative relationship between two connecting nodes. (*C*) A network cluster linked to cell survival and proliferation. The oval‐shaped and rectangular nodes are represented by gene and pathways, respectively. SOCS1 gene is a significant upstream regulator, which was upregulated in space as marked by red upward arrow. This regulator inhibits the JAK/STAT pathway as represented by the blunt headed edge between SOCS1 and JAK/STAT pathway. All the networks in this figure were inhibited in space as predicted by MAP. Their regulation status are shown by blue downward arrow. The arrow‐headed edges between the networks represent the activating relationships. (*D*) Hierarchical clustering of DEGs linked to the networks contributing to cell survival and proliferation. The genes enriching at least two of the five networks are clustered by Euclidean calculations. The color code of the gene regulation is in the left bottom. Deep red and blue colors represent upregulation and downregulation, respectively, whereas yellow represent no change. DEG = differentially expressed gene; DEMs = differentially expressed metabolites; IPA = Ingenuity Pathway Analysis; MAP = molecule activity predictor.

The canonical network analysis prompted us to construct six de novo biological networks, which potentially play key roles in spaceflight‐induced muscle loss, namely (i) protein metabolism, (ii) lipid metabolism, (iii) carbohydrate metabolism, (iv) muscle health, (v) inflammation, and (vi) mitochondrial dysfunction (Fig. 2*A*, Table S2). All of these networks were significantly enriched by the hypergeometric test (*p* < .05). Significantly enriched subnetworks (*p* < .05) linked to these individual noncanonical networks were mined and their regulation status (activated versus inhibited) were computed by molecule activity predictor (MAP) in IPA.

The protein metabolism network was enriched by 113 DEGs and seven DEMs that co‐constructed three key subnetworks: protein synthesis, catabolism, and translation; all three were predicted to be inhibited in FLT mice by the MAP algorithm. The carbohydrate metabolism network was enriched by 105 DEGs and 12 DEMs that co‐enriched the subnetworks, such as the uptake and quantity of carbohydrate, glucose (all inhibited in FLT), and glucose metabolism disorder network (activated in FLT). Enriched by 119 DEGs and 28 DEMs, a lipid metabolism network was built upon nine subnetworks including lipid synthesis and concentrations; both were inhibited in FLT mice as per our in silico prediction.

We curated 108 DEGs and three DEMs that are directly linked to muscle functions. Its subnetworks such as muscle formation, contraction, and differentiation were inhibited, but the proliferation network of the muscle cell was activated in FLT as per the MAP algorithm. In the context of muscular contraction, we further investigated the gene level activities of the calcium channel: they are composed of 28 DEGs and 17 DEMs (Table S2 *D*), and its subnetworks linked to flux and calcium ion quantity were inhibited in FLT in comparison to CTR. The inflammation network was enriched by 114 DEGs and 39 DEMs. This overarching network assembled a large set of inhibited subnetworks in spaceflight that included the activation and proliferation of lymphocytes, leukocytes, and phagocytes, and the synthesis of nitric oxide. Finally, the network linked to mitochondrial function was enriched by 42 DEGs and 18 DEMs (Fig. 2*B*). Its subnetworks included synthesis and hydrolysis of ATP, generation of reactive oxygen species and nitric oxide, and mitochondrial permeability. All of these subnetworks were inhibited in FLT mice as predicted by the MAP algorithm.

Table 2 enlists six upstream regulators; the regulation status (activated versus inhibited) of the cluster of DEGs and DEMs controlled by each regulator is quantified by the *Z*‐score. Genes STAT5B, SMAD3, and HSF1 encode transcription‐regulating proteins. Monoethylhexyl phthalate (MEHP) is a chemical toxicant, which was upregulated in FLT and potentially inhibited a cluster of 16 DEGs. SOCS1 is a downregulated gene in FLT and one of the top‐ranked upstream inhibitors regulating nine DEGs and one DEM. Table S3 enlisted those differentially expressed genes, which are controlled by these upstream regulators.

**Table 2:** List of Upstream Regulators of Interest

Upstream regulator	Name	Log_2_(fold change)	*Z*‐score	*p*	Molecules to regulate (gene/metabolite)
SOCS1	Suppressor of cytokine signaling 1	2.02	−2.14	.02	9/1
STAT5B	Signal transducer and activator of transcription 5B	0.64	1.70	5.52E‐10	33/1
SMAD3	Mothers against decapentaplegic homolog 3	0.50	1.86	.05	12/3
HSF1	Heat shock transcription factor 1	0.40	−1.67	3.12E‐05	19/1
MEHP	Mono‐(2‐ethyl hexyl) phthalate	0.25	−2.50	2.70E‐04	16/0
PLAU	Plasminogen activator	−0.84	−1.67	.04	5/1

These upstream regulators are sorted based on their fold changes (FLT/CTR) transformed to log scale base 2.

## Discussion

Spaceflight is an environment encompassing a set of atypical stress components such as weightlessness or microgravity, low‐dose radiation, and disrupted circadian rhythm. Recent studies further linked spaceflight to immune deficiencies, metabolic syndrome, bioenergetics dysfunction, and vascular remodeling.^(^ ^14^, ^15^, ^16^, ^17^, ^18^ ^)^ Each of these factors individually has shown detrimental effects on musculoskeletal health; in this perspective, this Rodent Research 4 (RR4) project presented us a unique opportunity to investigate the cumulative effects of all of these stress factors on muscle fitness. Our previous studies^(^ ^11^, ^19^ ^)^ showed that femoral and tibial trabecular bone volume was significantly reduced during spaceflight. Supplementing this knowledge, we reported the reduction of the wet mass of quadriceps, EDL, and soleus muscles in FLT compared to CTR mice. Extending previous reports,^(^ ^3^, ^4^, ^5^, ^6^ ^)^ present multiomics investigations targeted quadriceps, a major weight‐bearing muscle. Furthermore, the larger biomass of quadriceps in comparison to soleus and EDL was proportionate to the demand of the tissue size needed for multiomics undertakings.

A small but mounting number of studies have been focused on spaceflight‐related muscular atrophy; however, these results have often failed to capture the comprehensive picture due to the lack sufficient sample size^(^ ^20^ ^)^ and targeted or localized approaches.^(^ ^7^ ^)^ Bridging the gap, we used 10 mice/group for phenotypic observation and a randomly selected four mice/group were investigated for large‐scale transcript sequencing‐metabolite regulations. Systems integration of gene‐metabolite profiles have emerged as a successful approach to understand the temporal profile of a series of molecular events.^(^ ^13^, ^21^ ^)^

Overall, our observations revealed that the genes promoting muscle atrophy and protein degradation^(^ ^22^, ^23^ ^)^ were upregulated in space, while those genes which encode proteins linked to muscle fiber differentiation,^(^ ^20^, ^22^, ^24^ ^)^ calcium channel operation,^(^ ^23^ ^)^ and muscular functions^(^ ^23^, ^26^ ^)^ became downregulated. In particular, spaceflight inhibited or altered the expression of the genes and proteins related to the myosin heavy chain (MYH) family that typically contributes to the formation and function of skeletal muscle,^(^ ^27^ ^)^ and previously identified as the marker of compromised muscular functions triggered by spaceflight.^(^ ^28^, ^29^ ^)^ Our mRNA deep‐sequencing approach showed reduced expressions of four genes related to myosin and another three related to troponin in the FLT group compared to the CTR group. A potential negative impact of spaceflight on sarcomere architecture was further highlighted by the inhibited signaling of the integrin and actin cytoskeleton that typically provides the structural stability of muscle^(^ ^30^ ^)^ and facilitates muscular contraction,^(^ ^31^ ^)^ respectively. In this context, the inhibited molecular network in FLT linked to the operation of the calcium ion channel is worth noting. Therefore, it is conceivable that the FLT mice would have poorly functioning, or a reduced quantity of contractile units, in their skeletal muscle fibers. However, we could not measure sarcomeres from our mice because of tissue preparation limitations during spaceflight. Additionally, our study is limited by not recording body weights of the mice. Measuring body weights in spaceflight was infeasible. We decided against weighing the frozen carcasses of mice on the ground for several reasons. First, the possible differential amount of liquid crystallization in the carcass could confound the result. Second, the amount of blood withdrawn by cardiac puncture by the astronauts varied significantly from mouse to mouse. Third, half of the mice had their hindlimb removed. Finally, our first priority was to rapidly extract the tissues to minimize the freeze‐thaw cycle. Nevertheless, previous studies reported insignificant change in body weights of mice after space travels^(^ ^32^, ^33^ ^)^ and after exposing mice to hindlimb unloading on the ground.^(^ ^34^ ^)^

Altered calcium usage, decreased mtDNA CNs, and inhibited tricarboxylic acid (TCA) cycles are likely to converge to impair mitochondrial function, because these are causally related to essential cellular metabolism.^(^ ^35^, ^36^ ^)^ The mitochondrial dysfunction in space could explain the dysregulation occurring at the level of the sarcomeres that ultimately contributes to significant muscle loss. Typically, the muscular efficiency and endurance is dependent on carbohydrate metabolism, which is supported by the fatty acid metabolism as the secondary source of energy, while anaerobic glycolysis provides energy during intensive activities.^(^ ^37^, ^38^ ^)^ Our gene‐metabolite integrative analysis revealed that all of these energy sources, required for proper muscular functions, were inhibited in the spaceflight, potentially driving the host to a major energy‐deprived state.^(^ ^39^ ^)^

This energy‐deprived condition could be associated with the impairment of additional energy‐expensive mechanisms. One foremost energy‐expensive mechanism, namely the host’s immune response, was associated with a list of significantly inhibited canonical networks in FLT mice having Janus kinase (JAK)/signal transducer and activator of transcription (STAT) signal at the hub of C‐X‐C chemokine receptor type 4 (CXCR‐4)‐signaling, FGF‐signaling, NGF‐signaling, and STAT3‐signaling networks.^(^ ^40^ ^)^ Immunodeficiency in FLT mice was further highlighted as the majority of upstream regulators that encode proteins to act at the immunity checkpoints (ie, SOCS1^(^ ^41^ ^)^ and SMAD3^(^ ^42^ ^)^) or to mediate mitochondrial operations (ie, MEHP^(^ ^43^ ^)^). Given the fact that SOCS1 is an established negative regulator of the JAK/STAT network,^(^ ^42^ ^)^ and hence its downstream pathways,^(^ ^40^ ^)^ spaceflight appears to inhibit a large cascade of molecular networks which compromise the immune fitness by upregulating SOCS1 gene expression. The relevant network cluster regulated by SOCS1^(^ ^45^ ^)^ is displayed in Fig. 2*C* and hierarchical clustering of the DEGs enriching this network cluster is shown in Fig. 2*D*.

However, the question remains as to whether the disuse is the cause or consequence of shifting the expressions of those molecules, which promotes energy deprivation. In either case, it is also notable that our data on the immune functions in spaceflight correlated well with previous works that have associated these immunodeficiencies to energy deprivation in space.^(^ ^12^, ^16^, ^46^ ^)^ It is known that oxidative stress and inflammation have a negative impact on muscle health and contribute to sarcopenia,^(^ ^47^, ^48^ ^)^ and it has been speculated that to mitigate this damage, cells downregulate metabolism and immune functions that typically produce reactive oxygen species.^(^ ^49^ ^)^

At the background of the rampant negative consequences of spaceflight on healthy rodent quadriceps, there are potentially certain mitigation strategies in effect, again vital information, namely the initiation time of these biofunctions, remained obscure. Activation of the EIF2 network is one such compensating process that facilitates protein synthesis,^(^ ^50^ ^)^ controls mitochondrial dysfunction,^(^ ^51^ ^)^ and promotes the myogenic program.^(^ ^52^ ^)^ In concert, STAT5B, a promoter of muscle growth,^(^ ^53^ ^)^ was found to be upregulated, and significantly activated, as an upstream regulator. A number of genes related to sarcomere architecture are regulated by STAT5B, which by a recent account was shown to facilitate the healing cascade^(^ ^54^ ^)^; hence, this molecule could be a promising therapeutic target.

In conclusion, we report a comprehensive genome‐to‐phenome picture of murine quadriceps that have been subjected to ~4 weeks of disuse in spaceflight. Energy deprivation was found at the crossroads of several health deficiencies that have long been linked to spaceflight, such as muscular atrophy, metabolic impairment, and immunodeficiency. Causal relationships between these biofunctions are likely to be achieved in the near future because the process of expanding animal handling capability is underway at the ISS.

## Disclosures

Authors have no conflict of interest.

## Peer Review

The peer review history for this article is available at https://publons.com/publon/10.1002/jbmr.4102.

## Supporting information


**Supplementary Data**

**Figure S1.** Quadriceps samples’ quality control check. Post TMM normalization, the normalized counts of individual samples were plotted in the box and whisker plot. Here the box covers the interquartile range (from Q1 to Q3), the middle line across the box represents the mean value and the two ends of the whisker represents the range (from maximum to minimum). The plot shows that TMM normalization achieved a significant homology across the samples from FLT and CTR.

**Table S1.** The list of canonical pathways significantly perturbed by differentially expressed genes and metabolites. The list is sorted based on the individual pathway’s z‐score. Pathways that scored a positive z‐score above 1.5 were considered activated; while those pathways, which scored a negative z‐score lower than −1.5 were considered inhibited. There are two activated and 21 inhibited pathways listed in this table. The log transformed p‐values, number of molecules enriching particular pathway and the molecular IDs (gene symbol and metabolite name) are listed as well.
**Table S2**. The noncanonical networks linked to musculoskeletal operations. Each table includes the gene symbol and gene name, and sorted based on their fold changes transformed to log scale base 2. (A) Protein metabolism network was enriched by 44 upregulated and 53 downregulated biomolecules. The biomolecules included 113 genes and 7 metabolites. (B) Carbohydrate metabolism network was enriched by 47 upregulated and 70 downregulated biomolecules. The biomolecules included 105 genes and 12 metabolites. (C) Lipid metabolism network was enriched by 49 upregulated and 98 downregulated biomolecules. The biomolecules included 119 genes and 28 metabolites. (D) Muscle function network was enriched by 51 upregulated and 60 downregulated biomolecules. The biomolecules included 108 genes and 3 metabolites. The biomolecules included 108 genes and 3 metabolites. The molecules linked to calcium ion (Ca^+2^) channel are highlighted. (E) Inflammation network was enriched by 64 upregulated and 89 downregulated biomolecules. The biomolecules included 114 genes and 39 metabolites. (F) Mitochondrial dysfunction network was enriched by 18 upregulated and 40 downregulated biomolecules. The biomolecules included 42 genes and 16 metabolites.
**Table S3**. The list of genes associated with the upstream regulators of interest, namely SOCS1, STAT5B, SMAD3, HSF1, mono‐(2‐ethylhexyl) phthalate and PLAU. For individual upstream regulator, the gene symbols were sorted in descending order.
