# Supplementary material for: Gene‐Metabolite Network Linked to Inhibited Bioenergetics in Association With Spaceflight‐Induced Loss of Male Mouse Quadriceps Muscle
Source: J Bone Miner Res. 2020 Jul 30;35(10):2049–57. doi: 10.1002/jbmr.4102 (PMC7689867; doi:10.1002/jbmr.4102)
Supplement: Supplementary file 1 — Supplementary Data [file JBMR-35-2049-s001.pdf]

Supplementary data

Materials and Methods

Male C57BL/6j of ~7 weeks of age were purchased from Jackson Laboratory, ME and shipped to Kennedy Space Center (KSC), FL. The animals were ear punched for identification. A randomly selected cage of ten mice were launched into space on Space X-10, February 19, 2017. This cohort was labeled as FLT. Five days later these mice reached to International Space Station (ISS) and henceforth housed in the vivarium of ISS. With a five day offset, the ground control (CTR) ran in parallel and were comprised of another 10 age-matched male mice. We introduced a 5 day lag between FLT and CTR studies to spread the workload and to allow for matched ground controls in terms of experimental conditions such as temperature changes etc. NASA rodent research habitat (https://www.nasa.gov/ames/research/space-biosciences/rodent-research-hardware) was used for both ground and space study with same diet and light-dark cycle. The animal habitat used for both spaceflight (FLT) and ground control (CTR) has been described previously (1). Both set of mice (FLT and CTR) were housed in a similar caging condition including standard light-dark cycle with same diet and water *ad lib*. Mice were euthanize around 4 weeks post launch on the ISS and on Earth (asynchronous by 5 days). At the end of respective studies, animals were primed by the injection of ketamine/xylazine (150/45 mg/kg) to anesthetize the mice and cervical dislocation was conducted to complete the euthanasia. The endpoint was confirmed by typical procedures like pinching etc. before the carcasses were snap frozen at -80°C. Figure 1A shows the timeline followed by this project named Rodent Research 4 (RR4) jointly supported by National Aeronautics and Space Administration (NASA), The Center for the Advancement of Science in Space (CASIS) and Department of Defense (DoD). The frozen samples were returned to Earth; upon retrieval, the FLT and CTR samples were gathered at USACEHR, MD and all samples were processed at the same time following a protocol described earlier(1, 2). Each carcass was dissected to collect 161 tissue samples within 20 minutes from its removal from a freezer. The muscle tissues were isolated and frozen at -80°C for further analysis.

At the time of biomolecular extraction, the quadriceps tissues were first weighed and then cryogenically homogenized using a Cryomill (Retsch GmbH, Germany). An aliquot of the homogenates was allotted for extraction RNA/DNA using the standard TRIzol (Invitrogen, Thermo Fisher Scientific, Wilmington, MA) method.

DNA extracted from the interphase of TRIzol solution was assayed to measure the copy number variation (CNV) of mitochondrial DNA (mtDNA) using the NovaQUANT™ Mouse Mitochondrial to Nuclear DNA Ratio Kit (Novagen, WI). Vendor provided PCR primer pairs were used to amplify two mitochondrial genes (trLEV and 12s RNA) in Applied Biosystems 7900HT real-time qPCR. CNV was calculated following the equation:

$$mtDNA\ CNV_{FLT-CTR} = \left(\frac{2^{Ct[BECN1]-Ct[trLEV]} + 2^{Ct[NEB]-Ct[12s]}}{2}\right)_{FLT} - \left(\frac{2^{Ct[BECN1]-Ct[trLEV]} + 2^{Ct[NEB]-Ct[12s]}}{2}\right)_{CTR}$$

Here Ct stands for the PCR cycle number. BECN1 and NEB are the two markers of mitochondrial DNA. Likewise, trLEV and 12s are the markers of nuclear DNA (nDNA).

The aqueous phase of TRIzol solution was extracted and ethanol was added to provide optimal binding conditions for all RNA molecules. The sample was then applied to the RNeasy Mini spin column per manufacturer's instructions. The total RNA was eluted in RNase-free water and quality control checks were performed using NanoDrop spectrophotometer (Thermo Fisher Scientific, Wilmington, MA) and Agilent 4200 TapeStation (Agilent Technologies, Santa Clara, CA). Average RIN values± standard deviation (Avg±STD) of FLT and CTR samples were 4.48±1.74 and 5.36±0.96, respectively; Avg±STD of 260/280 values of FLT and CTR were 1.43±0.31 and 1.82±0.12, respectively; and Avg±STD of total concentration of FLT and CTR samples were 383.3±111.1 ng/ul and 522.6±162.0 ng/ul, respectively.

The sequencing library preparation was carried out using 500 ng of total RNA input with the TruSeq Stranded mRNA Sample Preparation Kit (Illumina Inc, San Diego, CA) following the manufacturer's procedures. In brief, total RNA was fragmented using divalent cations under elevated temperature. Cleaved RNA fragments were then converted to first strand cDNA using reverse transcriptase SuperScript II (Thermo Fisher, Waltham, MA). Strand selection was done by replacing dTTP with dUTP followed by second strand cDNA synthesis using DNA Polymerase I and RNase H (Illumina Inc, San Diego, CA). A tail was added to the 3' ends of the blunt fragments. Unique indexing adapters were ligated to the ends of the ds cDNA fragments and the products were enriched with PCR and purified to create the final cDNA library. The quality of the libraries were determined using the on D1000 ScreenTape on the Agilent TapeStation 2200 (Agilent Technologies, Santa Clara, CA, USA). Libraries were quantified with real-time quantitative PCR using KAPA SYBR® FAST Universal qPCR Kit (Kapa Biosystems). Next, the libraries were normalized, pooled, and quantified using the dsDNA HS Kit on the Qubit 3.0 Fluorometer ; followed by sequencing 2x150 cycles paired-end reads on an Illumina HiSeq 4000 platform (Illumina, Inc., San Diego, CA) using the Illumina HiSeq 3000/4000 SBS kit following the manufacturer's instructions.

mRNA raw reads were quality filtered with Q score above 30 and adaptors were trimmed using Trimmomatic v0.38 and reads were further aligned using STAR aligner v2.6.0 against UCSC genome assembly for Mus Musculus mm10. Gene counts were further filtered using counts per million (CPM) cutoff greater than 0 across eight samples. Counts were normalized using the TMM normalization method using edgeR package v3.24.3. The library depth and count

distribution was plotted in Figure S2. Differential expression was calculated by fitting a negative binomial generalized log-linear model to the read counts for each gene using the limma package v3.38.3.

The second aliquot of the homogenized quadriceps tissues was re-suspended in ice cold Phosphate-buffered saline (PBS) with a protease inhibitor tablet. The resuspension was centrifuged at 5000x g for 5 minutes and the supernatant was collected. The load of myosin protein was estimated by using the Abbexa (Cambridge, UK) Mouse Myosin ELISA kit (Catalog# ABX350887-96RXN) and the optical density at 450nm (OD450) was measured by BMG Labtech microplate reader, FLUOstar Omega series.

A third aliquot of quadriceps homogenates was used for global metabolomics analysis using mass spectrometry performed on a Q-TOF Premier mass spectrometer (Waters, Inc.). The metabolite profiling of muscle homogenate was conducted as per the report published previously (3, 4). 50 mg of the homogenized tissue was dissolved in chilled extraction buffer (50% methanol in water). The protein was precipitated by addition of equal volumes of acetonitrile; samples were centrifuged. 5 ul of the sample was injected onto a reverse-phase 50 × 2.1mm Acquity 1.7-μm C18 column (Waters Corp, Milford, MA) using an Acquity UPLC system (Waters) with a gradient mobile phase consisting of 2% acetonitrile in water containing 0.1% formic acid (Solvent A) and 2% water in acetonitrile containing 0.1% formic acid (Solvent B) and resolved for 10 min at a flow rate of 0.5 mL/min. The gradient consisted of 100% A for 0.5 min with a ramp of curve 6–100% B from 0.5 to 10 min. The column eluent was introduced directly into the mass spectrometer by electrospray. Mass spectrometry was performed on a Q-TOF Premier mass spectrometer (Waters) operating in either positive-ion or negative-ion electrospray ionization (ESI+/−) mode with a capillary voltage of 3200 V and a sampling cone voltage of 20 V in negative mode and 35 V in positive mode. The desolvation gas flow was set to 800 L/h and the temperature was set to 350 °C. The cone gas flow was 25 L/h, and the source temperature was 120 °C. Accurate mass was maintained by introduction of LockSpray interface of sulfadimethoxine (311.0814 [M+H] + or 309.0658 [M−H]−) at a concentration of 250 pg/μL in 50% aqueous acetonitrile and a rate of 150 μL/min. Data were acquired in centroid mode from 50 to 850m/z in MS scanning. Centroided and integrated mass spectrometry data obtained in duplicates from the UPLC-TOFMS were processed using CEU Mass Mediator database (www.ceumass.eps.uspceu.es).

Result

Quadriceps tissue specific sequencing data was able to map onto the whole mouse genome at the average rate of 20,000 reads per gene. As a result, we captured 10,874 genes (~54% of whole mouse genes) with reads more than 10 counts (counts per million > 0.5) in all 8 samples. In particular, 79.5% (range: 77.5%-81.5%) and 74.1% (range: 79.5%-65.5%) of FLT and CTR reads respectively were mapped to mouse genome. We used these 10,874 mouse genes to curate 776 differentially expressed genes (DEG) between FLT and CTR. This DEG list included 378 up regulated and 398 down regulated genes; 19 of this 776 genes met False Discovery Rate (FDR) 0.1, while 14 genes met the FDR cutoff 0.05. Mass spectrometry analysis of quadriceps tissues identified 740 differentially expressed chemicals (DECs) between FLT and CTR; which included 440 downregulated and 300 upregulated metabolites. None of these metabolites met FDR correction.

Functional analysis of DEGs and DECs were seeded to identify 23 significantly enriched (scored by hypergeometric p values) and significantly regulated (measured by Z-score) canonical networks. Table S1 listed all significantly regulated canonical networks. Taking lead from the canonical network analysis, we constructed 6 non-canonical networks, which are enlisted in Table S2.

Upstream regulators defined as any molecular entity that is known to affect the expression of a single gene or a family of genes via direct or indirect causal association(5). Upstream Regulator Analysis (URA) in the Ingenuity Pathway Analysis (IPA) platform was adapted from the Connectivity Map tool(6), where the relationships among the genes were mapped using database developed by literature curation. These upstream regulators could have significant clinical potential, since a large number of genes and their biofunctions can be systematically controlled via targeting single upstream regulator. Table S3 listed the genes regulated by the upstream regulators of interest.

Figure legends:

Figure S1. Quadriceps samples' quality control check. Post TMM normalization, the normalized counts of individual samples were plotted in the box and whisker plot. Here the box covers the interquartile range (from Q1 to Q3), the middle line across the box represents the mean value and the two ends of the whisker represents the range (from maximum to minimum). The plot shows that TMM normalization achieved a significant homology across the samples from FLT and CTR.

Table legends:

Table S1. The list of canonical pathways significantly perturbed by differentially expressed genes and metabolites. The list is sorted based on the individual pathway's z-score. Pathways that scored a positive z-score above 1.5 were considered activated; while those pathways, which scored a negative z-score lower than -1.5 were considered inhibited. There are two activated and twenty one inhibited pathways listed in this table. The log transformed p-values, number of molecules enriching particular pathway and the molecular IDs (gene symbol and metabolite name) are listed as well.

Table S2. The non-canonical networks linked to musculoskeletal operations. Each table includes the gene symbol and gene name, and sorted based on their fold changes transformed to log scale base 2. (A) Protein metabolism network was enriched by 44 upregulated and 53 downregulated biomolecules. The biomolecules included 113 genes and 7 metabolites. (B) Carbohydrate metabolism network was enriched by 47 upregulated and 70 downregulated biomolecules. The biomolecules included 105 genes and 12 metabolites. (C) Lipid metabolism network was enriched by 49 upregulated and 98 downregulated biomolecules. The biomolecules included 119 genes and 28 metabolites. (D) Muscle function network was enriched by 51 upregulated and 60 downregulated biomolecules. The biomolecules included 108 genes and 3 metabolites. The biomolecules included 108 genes and 3 metabolites. The molecules linked to calcium ion (Ca^{+2}) channel are highlighted. (E) Inflammation network was enriched by 64 upregulated and 89 downregulated biomolecules. The biomolecules included 114 genes and 39 metabolites. (F) Mitochondrial dysfunction network was enriched by 18 upregulated and 40 downregulated biomolecules. The biomolecules included 42 genes and 16 metabolites.

Table S3. The list of genes associated with the upstream regulators of interest, namely SOCS1, STAT5B, SMAD3, HSF1, mono-(2-ethylhexyl) phthalate and PLAU. For individual upstream regulator, the gene symbols were sorted in descending order.

References

1. Dadwal UC*, et al.* (2019) The effects of spaceflight and fracture healing on distant skeletal sites. *Scientific reports* 9.
2. Maupin KA*, et al.* (2019) Skeletal adaptations in young male mice after 4 weeks aboard the International Space Station. *npj Microgravity* 5(1):1-10.
3. Chakraborty N*, et al.* (2018) Gene-metabolite profile integration to understand the cause of spaceflight induced immunodeficiency. *NPJ Microgravity* 4:4.
4. Gautam A*, et al.* (2018) Altered fecal microbiota composition in all male aggressor-exposed rodent model simulating features of post-traumatic stress disorder. *Journal of neuroscience research* 96(7):1311-1323.
5. Kramer A, Green J, Pollard J, Jr., & Tugendreich S (2014) Causal analysis approaches in Ingenuity Pathway Analysis. *Bioinformatics* 30(4):523-530.
6. Lamb J*, et al.* (2006) The Connectivity Map: using gene-expression signatures to connect small molecules, genes, and disease. *Science* 313(5795):1929-1935.
