# Supplementary figures and images for: Gene‐Metabolite Network Linked to Inhibited Bioenergetics in Association With Spaceflight‐Induced Loss of Male Mouse Quadriceps Muscle
Source: J Bone Miner Res. 2020 Jul 30;35(10):2049–57. doi: 10.1002/jbmr.4102 (PMC7689867; doi:10.1002/jbmr.4102)

Figure S1

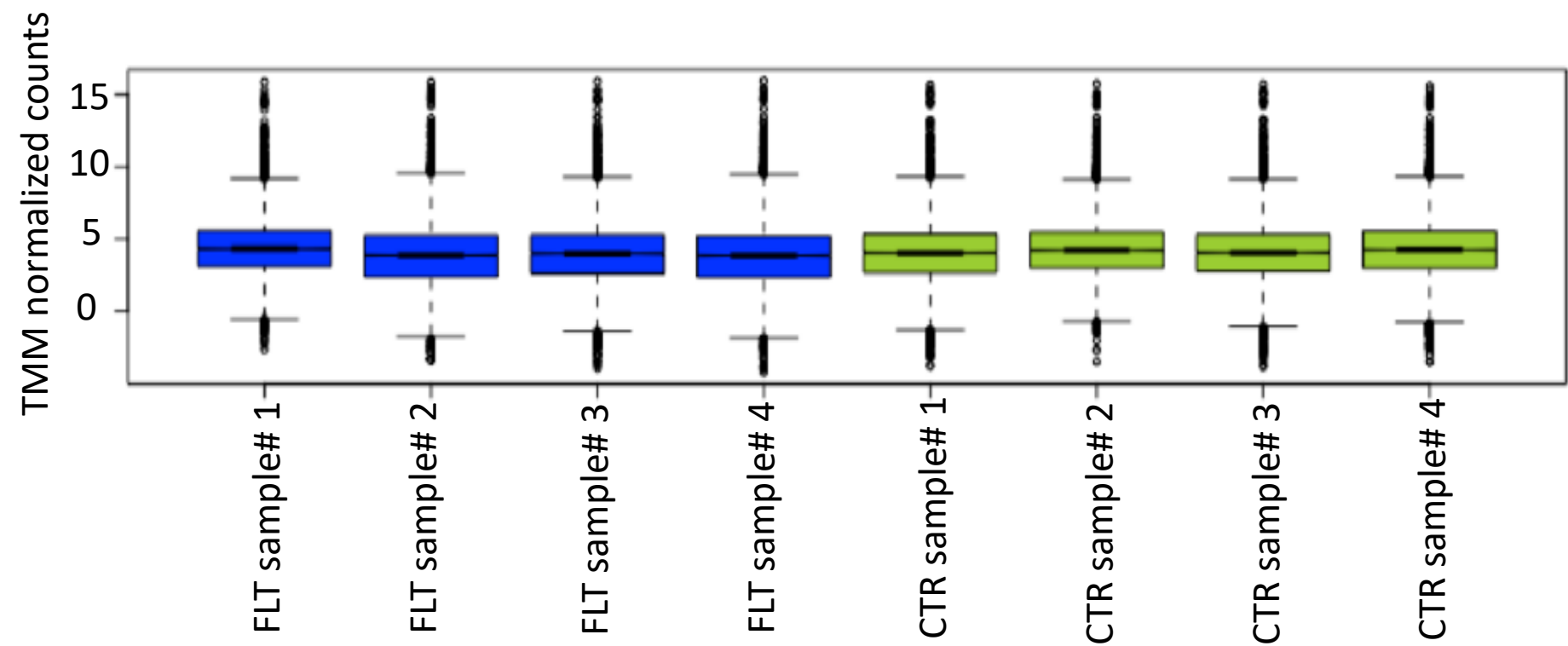

Supplement: Supplementary file 2 — Figure S1. Quadriceps samples’ quality control check. Post TMM normalization, the normalized counts of individual samples were plotted in the box and whisker plot. Here the box covers the interquartile range (from Q1 to Q3), the middle line across the box represents the mean value and the two ends of the whisker represents the range (from maximum to minimum). The plot shows that TMM normalization achieved a significant homology across the samples from FLT and CTR. [file JBMR-35-2049-s002.pdf]
