# Supplementary material for: Gene‐Metabolite Network Linked to Inhibited Bioenergetics in Association With Spaceflight‐Induced Loss of Male Mouse Quadriceps Muscle
Source: J Bone Miner Res. 2020 Jul 30;35(10):2049–57. doi: 10.1002/jbmr.4102 (PMC7689867; doi:10.1002/jbmr.4102)
Supplement: Supplementary file 3 — Table S1. The list of canonical pathways significantly perturbed by differentially expressed genes and metabolites. The list is sorted based on the individual pathway’s z‐score. Pathways that scored a positive z‐score above 1.5 were considered activated; while those pathways, which scored a negative z‐score lower than −1.5 were considered inhibited. There are two activated and 21 inhibited pathways listed in this table. The log transformed p‐values, number of molecules enriching particular pathway and the molecular IDs (gene symbol and metabolite name) are listed as well. Table S2. The noncanonical networks linked to musculoskeletal operations. Each table includes the gene symbol and gene name, and sorted based on their fold changes transformed to log scale base 2. (A) Protein metabolism network was enriched by 44 upregulated and 53 downregulated biomolecules. The biomolecules included 113 genes and 7 metabolites. (B) Carbohydrate metabolism network was enriched by 47 upregulated and 70 downregulated biomolecules. The biomolecules included 105 genes and 12 metabolites. (C) Lipid metabolism network was enriched by 49 upregulated and 98 downregulated biomolecules. The biomolecules included 119 genes and 28 metabolites. (D) Muscle function network was enriched by 51 upregulated and 60 downregulated biomolecules. The biomolecules included 108 genes and 3 metabolites. The biomolecules included 108 genes and 3 metabolites. The molecules linked to calcium ion (Ca+2) channel are highlighted. (E) Inflammation network was enriched by 64 upregulated and 89 downregulated biomolecules. The biomolecules included 114 genes and 39 metabolites. (F) Mitochondrial dysfunction network was enriched by 18 upregulated and 40 downregulated biomolecules. The biomolecules included 42 genes and 16 metabolites. Table S3. The list of genes associated with the upstream regulators of interest, namely SOCS1, STAT5B, SMAD3, HSF1, mono‐(2‐ethylhexyl) phthalate and PLAU. For individual upstream regul [file JBMR-35-2049-s003.pdf]

Table S1. Cannonical networks co-enriched by gene and metabolites. The list is sorted in the descending order of z-score

Canonical Pathways	-log(p-value)	z-score	Number of molecules	Molecules
EIF2 Signaling	19.90	2.75	44	RPL11,RPL24,EIF2AK4,MAPK1,RPS18,EIF4A2,HSPA5,EIF4E,FGFR3,RPS28,RPL14,RPL27A,RPL35,RPL18A,RPL21,EIF3A,RPL36,RPL18,RPL31,MAP2K1,EIF1AY,EIF2AK1,FGFR1,EIF5B,RPS8,RPL17,EIF4G3,RPS21,RPL37A,RAP1A,RPS29,RPL28,EIF3M,RPS15,PTPN11,RPS26,PPP1R15A,RPS15A,RPL37,RPS25,RPL13A,EIF3L,RPSA,EIF3K
D-myo-inositol-5-phosphate Metabolism	3.65	1.50	16	SOCS3,ATP,PPP1R1A,ADP,MDP1,NT5C,STYX,MTMR6,CA3,PLCD3,PTPN11,PPP1R7,DUSP13,DUSP10,PLCD4,PTPN22
Prolactin Signaling	3.45	-1.51	11	FGFR3,FOS,SOCS3,SOCS1,PTPN11,MAPK1,FGFR1,JAK2,STAT5B,MAP2K1,RAP1A

ILK Signaling	4.27	-1.61	19	MYH4,PXN,FN1,MAPK1,MYL2,PPP2CA,ACTN2,FGFR1,MYH7,MYH7B,FGFR3,FOS,MYH2,RHOQ,CFL2,PTPN11,RHOB,MYL4,MYH1
Neuregulin Signaling	1.30	-1.63	7	MAPK1,PTPN11,ERRFI1,CRK,STAT5B,MAP2K1,RAP1A
ErbB4 Signaling	1.66	-1.63	7	FGFR3,YAP1,MAPK1,PTPN11,FGFR1,MAP2K1,RAP1A
TCA Cycle II (Eukaryotic)	2.55	-1.63	6	SDHA,SUCLA2,ATP,ADP,DLD,SDHD
Pyrimidine Ribonucleotides Interconversion	3.29	-1.63	8	SMARCA1,NUDT18,ATP,ENTPD6,ADP,CMPK1,PCK1,ZRANB3
NGF Signaling	1.45	-1.67	9	FGFR3,PTPN11,MAPK1,FGFR1,TRIO,CRK,MAP2K1,RAP1A,MAP3K3
Renin-Angiotensin Signaling	1.68	-1.67	10	FGFR3,FOS,ADCY2,ATP,PTPN11,MAPK1,FGFR1,JAK2,MAP2K1,RAP1A
Role of NANOG in Mammalian Embryonic Stem Cell Pluripotency	1.87	-1.67	10	FGFR3,IL6ST,PTPN11,MAPK1,FGFR1,WNT4,JAK2,FOXD3,MAP2K1,RAP1A
IL-8 Signaling	1.58	-1.73	13	FGFR3,FOS,RHOQ,MYL2,PTPN11,RHOB,MAPK1,FGFR1,ITGAV,MAP2K1,RAP1A,VASP,IRAK2

STAT3 Pathway	3.74	-1.73	14	IL6ST,SOCS1,SOCS3,IL4R,MAPK1,FGFR1,JAK2,RAP1A,FGFR3,PIM1,NTRK3,CDKN1A,CISH,MAP2K1
CXCR4 Signaling	3.34	-1.81	16	ADCY2,PXN,ATP,MAPK1,MYL2,FGFR1,CRK,RAP1A,FGFR3,FOS,RHOQ,PTPN11,RHOB,MYL4,MAP2K1,MYL12A
Oxidative Phosphorylation	4.88	-1.81	15	SDHA,NDUFS1,NDUFB7,COX5A,SDHD,NDUFB6,UQCRFS1,NDUFA3,ATP5S,ATP5PB,ATP5MC3,COX6A2
Actin Cytoskeleton Signaling	5.73	-1.88	24	MYH4,PXN,FN1,MAPK1,MYL2,FGF9,ACTN2,FGFR1,RDX,TRIO,CRK,MYH7,SLC9A1,RAP1A,MYH7B,FGFR3,MYH2,CFL2,PTPN11,MYL4,PFN2,MAP2K1,MYH1,MYL12A
FGF Signaling	1.34	-1.89	7	FGFR3,MAPK1,PTPN11,FGFR1,FGF9,CRK,MAP2K1
Antiproliferative Role of Somatostatin Receptor 2	1.45	-1.89	7	FGFR3,MAPK1,PTPN11,FGFR1,CDKN1A,MAP2K1,RAP1A

Th1 Pathway	2.11	-1.90	11	FGFR3,SOCS3,SOCS1,NOTCH4,NFIL3,PTPN11,FGFR1,HLA-DQA1,NFATC2,JAK2,HLA-DRB5
JAK/Stat Signaling	4.90	-1.94	13	SOCS1,SOCS3,MAPK1,FGFR1,JAK2,RAP1A,FGFR3,FOS,PTPN11,CDKN1A,CISH,STAT5B,MAP2K1
NRF2-mediated Oxidative Stress Response	6.34	-2.11	23	GSTM1,MAPK1,GSTM5,DNAJB4,FGFR1,NQO1,HERPUD1,GCLC,DNAJA1,RAP1A,TXNRD1,BACH1,FGFR3,CUL3,FOS,GSTM2,PTPN11,CAT,JUND,DNAJA2,AOX1,DNAJB6,MAP2K1
Integrin Signaling	3.64	-2.36	19	PXN,MAPK1,MYL2,ACTN2,FGFR1,CRK,RAP1A,FGFR3,WIPF1,RHOQ,RHOB,PTPN11,ITGAV,PFN2,CAPN7,TSPAN4,MAP2K1,VASP,MYL12A

Table S2A. The non-canonical networks linked to musculoskeletal operations. Each table includes the gene symbol and gene name, and sorted based on their fold changes transformed to log scale base 2. (A) Protein metabolism network was enriched by 44 upregulated and 53 downregulated biomolecules. The biomolecules included 113 genes and 7 metabolites.

Gene symbol	Entrez Gene Name	Expr Log Ratio
SOCS1	suppressor of cytokine signaling 1	2.023
FOS	Fos proto-oncogene, AP-1 transcription factor subunit	1.918
CDKN1A	cyclin dependent kinase inhibitor 1A	1.558
ARNTL	aryl hydrocarbon receptor nuclear translocator like	1.531
IGF2BP2	insulin like growth factor 2 mRNA binding protein 2	1.504
SOCS3	suppressor of cytokine signaling 3	1.319
FST	follistatin	1.238
FAM83D	family with sequence similarity 83 member D	1.152
TP63	tumor protein p63	1.086
HDAC8	histone deacetylase 8	1.08
AURKA	aurora kinase A	1.047
PIM1	Pim-1 proto-oncogene, serine/threonine kinase	1.003
UBC	ubiquitin C	0.892
PPP1R15A	protein phosphatase 1 regulatory subunit 15A	0.883
MVK	mevalonate kinase	0.787
MMP15	matrix metallopeptidase 15	0.711
RCN3	reticulocalbin 3	0.678
FBXW7	F-box and WD repeat domain containing 7	0.664
RPL37	ribosomal protein L37	0.624
FAM20C	FAM20C, golgi associated secretory pathway kinase	0.61
MRPL23	mitochondrial ribosomal protein L23	0.596
TRIM63	tripartite motif containing 63	0.589
RPS26	ribosomal protein S26	0.585
USP13	ubiquitin specific peptidase 13	0.575
SDC4	syndecan 4	0.573
RPL28	ribosomal protein L28	0.571
ERRFI1	ERBB receptor feedback inhibitor 1	0.533
RARA	retinoic acid receptor alpha	0.532
IGFBP6	insulin like growth factor binding protein 6	0.522
MFGE8	milk fat globule-EGF factor 8 protein	0.518
EIF5B	eukaryotic translation initiation factor 5B	0.51
RPL11	ribosomal protein L11	0.506
RPL35	ribosomal protein L35	0.502
KLHL8	kelch like family member 8	0.5
RPL13A	ribosomal protein L13a	0.497
ANO8	anoctamin 8	0.493
SMAD3	SMAD family member 3	0.493
SAMD4A	sterile alpha motif domain containing 4A	0.486
RPL14	ribosomal protein L14	0.485
MAN1B1	mannosidase alpha class 1B member 1	0.484

RPS8	ribosomal protein S8	0.481
EIF3K	eukaryotic translation initiation factor 3 subunit K	0.478
RPL21	ribosomal protein L21	0.473
RPS29	ribosomal protein S29	0.461
RPS15	ribosomal protein S15	0.45
RPL18A	ribosomal protein L18a	0.445
EIF2AK1	eukaryotic translation initiation factor 2 alpha kinase 1	0.438
RPL24	ribosomal protein L24	0.437
RPL37A	ribosomal protein L37a	0.436
RPL18	ribosomal protein L18	0.434
PSMB3	proteasome subunit beta 3	0.428
RPL17	ribosomal protein L17	0.416
RPL27A	ribosomal protein L27a	0.41
RPS21	ribosomal protein S21	0.41
NEDD8	neural precursor cell expressed, developmentally down-regulated 8	0.4
TCOF1	treacle ribosome biogenesis factor 1	0.395
RPSA	ribosomal protein SA	0.394
RPS28	ribosomal protein S28	0.393
TGM2	transglutaminase 2	0.393
RPS15A	ribosomal protein S15a	0.385
RPL36	ribosomal protein L36	0.383
RPL31	ribosomal protein L31	0.375
AURKAIP1	aurora kinase A interacting protein 1	0.373
RPS18	ribosomal protein S18	0.367
MAPT	microtubule associated protein tau	0.364
RPS25	ribosomal protein S25	0.362
EIF4G3	eukaryotic translation initiation factor 4 gamma 3	0.361
L-alanine	--	-0.288
ATP	--	-0.347
HSPA5	heat shock protein family A (Hsp70) member 5	-0.359
PSMD14	proteasome 26S subunit, non-ATPase 14	-0.363
EIF3L	eukaryotic translation initiation factor 3 subunit L	-0.378
MAPK1	mitogen-activated protein kinase 1	-0.388
HERPUD1	homocysteine inducible ER protein with ubiquitin like domain 1	-0.401
CCT4	chaperonin containing TCP1 subunit 4	-0.41
RTN4	reticulon 4	-0.415
UBR2	ubiquitin protein ligase E3 component n-recognin 2	-0.428
EIF4E	eukaryotic translation initiation factor 4E	-0.447
PPP1R2	protein phosphatase 1 regulatory inhibitor subunit 2	-0.447
TARBP2	TARBP2, RISC loading complex RNA binding subunit	-0.456
CUL3	cullin 3	-0.485
EIF3M	eukaryotic translation initiation factor 3 subunit M	-0.506
niacinamide	--	-0.514
PTPN11	protein tyrosine phosphatase, non-receptor type 11	-0.515
cholesterol sulfate	--	-0.521
CAPN7	calpain 7	-0.528
USP25	ubiquitin specific peptidase 25	-0.533

UBE2G1	ubiquitin conjugating enzyme E2 G1	-0.544
PPP2CA	protein phosphatase 2 catalytic subunit alpha	-0.545
EIF3A	eukaryotic translation initiation factor 3 subunit A	-0.547
STX12	syntaxin 12	-0.549
SPCS1	signal peptidase complex subunit 1	-0.553
UCHL3	ubiquitin C-terminal hydrolase L3	-0.557
EIF4A2	eukaryotic translation initiation factor 4A2	-0.567
MAP2K1	mitogen-activated protein kinase kinase 1	-0.568
EIF2AK4	eukaryotic translation initiation factor 2 alpha kinase 4	-0.577
CANX	calnexin	-0.582
BAG3	BCL2 associated athanogene 3	-0.586
ERLEC1	endoplasmic reticulum lectin 1	-0.589
NGLY1	N-glycanase 1	-0.601
SOX17	SRY-box 17	-0.627
CRK	CRK proto-oncogene, adaptor protein	-0.645
FN1	fibronectin 1	-0.65
DLD	dihydrolipoamide dehydrogenase	-0.675
TPP2	tripeptidyl peptidase 2	-0.695
CAT	catalase	-0.761
CNOT7	CCR4-NOT transcription complex subunit 7	-0.812
GJA1	gap junction protein alpha 1	-0.821
saquinavir	--	-0.828
PLAU	plasminogen activator, urokinase	-0.838
PFN2	profilin 2	-0.847
UBR1	ubiquitin protein ligase E3 component n-recognin 1	-0.855
ATP1B1	ATPase Na+/K+ transporting subunit beta 1	-0.863
NAGLU	N-acetyl-alpha-glucosaminidase	-0.9
JAK2	Janus kinase 2	-0.911
L2HGDH	L-2-hydroxyglutarate dehydrogenase	-0.924
ADP	--	-0.935
CCT6A	chaperonin containing TCP1 subunit 6A	-1.21
PER3	period circadian regulator 3	-1.534
methylprednisolone	--	-1.542

Table S2B. The non-canonical networks linked to musculoskeletal operations. Each table includes the gene symbol and gene name, and sorted based on their fold changes transformed to log scale base 2. (B) Carbohydrate metabolism network was enriched by 47 upregulated and 70 downregulated biomolecules. The biomolecules included 105 genes and 12 metabolites.

Gene symbol	Entrez Gene Name	Expr Log Ratio
SOCS1	suppressor of cytokine signaling 1	2.023
RRAD	RRAD, Ras related glycolysis inhibitor and calcium channel regulator	1.755
PNPLA3	patatin like phospholipase domain containing 3	1.74
UCN2	urocortin 2	1.556
ARNTL	aryl hydrocarbon receptor nuclear translocator like	1.531
IGF2BP2	insulin like growth factor 2 mRNA binding protein 2	1.504
NEU3	neuraminidase 3	1.32
SOCS3	suppressor of cytokine signaling 3	1.319
NR1D1	nuclear receptor subfamily 1 group D member 1	1.137
ABCB1	ATP binding cassette subfamily B member 1	1.051
ACVR2B	activin A receptor type 2B	1.019
DYRK2	dual specificity tyrosine phosphorylation regulated kinase 2	0.966
PPP1R3C	protein phosphatase 1 regulatory subunit 3C	0.943
ENHO	energy homeostasis associated	0.935
ADCY2	adenylate cyclase 2	0.888
PPP1R15A	protein phosphatase 1 regulatory subunit 15A	0.883
CHST12	carbohydrate sulfotransferase 12	0.826
EGLN3	egl-9 family hypoxia inducible factor 3	0.727
FGFR1	fibroblast growth factor receptor 1	0.725
ACACA	acetyl-CoA carboxylase alpha	0.704
PBX1	PBX homeobox 1	0.704
SORT1	sortilin 1	0.691
Foxp1	forkhead box P1	0.626
PFKFB3	6-phosphofructo-2-kinase/fructose-2,6-biphosphatase 3	0.612
HS6ST1	heparan sulfate 6-O-sulfotransferase 1	0.609
VAC14	Vac14, PIKFYVE complex component	0.6
SLC9A1	solute carrier family 9 member A1	0.585
SDC4	syndecan 4	0.573
PRKAG3	protein kinase AMP-activated non-catalytic subunit gamma 3	0.565
TFEB	transcription factor EB	0.536
IL4R	interleukin 4 receptor	0.523
FMOD	fibromodulin	0.519
APOD	apolipoprotein D	0.516
B3GAT3	beta-1,3-glucuronyltransferase 3	0.507
FN3K	fructosamine 3 kinase	0.499
MIF	macrophage migration inhibitory factor	0.494
SMAD3	SMAD family member 3	0.493
BSG	basigin (Ok blood group)	0.49
BSCL2	BSCL2, seipin lipid droplet biogenesis associated	0.487

MAN1B1	mannosidase alpha class 1B member 1	0.484
PLCD4	phospholipase C delta 4	0.474
GRB10	growth factor receptor bound protein 10	0.441
SIK3	SIK family kinase 3	0.43
ALDOA	aldolase, fructose-bisphosphate A	0.406
HSF1	heat shock transcription factor 1	0.391
MDP1	magnesium dependent phosphatase 1	0.39
IL6ST	interleukin 6 signal transducer	0.359
pyruvaldehyde	--	-0.288
alanine	--	-0.288
ATP	--	-0.347
HSPA5	heat shock protein family A (Hsp70) member 5	-0.359
MAPK1	mitogen-activated protein kinase 1	-0.388
RAB14	RAB14, member RAS oncogene family	-0.395
SH3GLB1	SH3 domain containing GRB2 like, endophilin B1	-0.41
SRSF2	serine and arginine rich splicing factor 2	-0.411
acetaminophen	--	-0.424
CHPT1	choline phosphotransferase 1	-0.44
carnosine	--	-0.451
CXCL14	C-X-C motif chemokine ligand 14	-0.454
HADH	hydroxyacyl-CoA dehydrogenase	-0.476
P2RY2	purinergic receptor P2Y2	-0.477
SERINC3	serine incorporator 3	-0.49
RHOQ	ras homolog family member Q	-0.493
ST3GAL5	ST3 beta-galactoside alpha-2,3-sialyltransferase 5	-0.497
CPE	carboxypeptidase E	-0.507
PFKFB4	6-phosphofructo-2-kinase/fructose-2,6-biphosphatase 4	-0.514
PTPN11	protein tyrosine phosphatase, non-receptor type 11	-0.515
KCNC1	potassium voltage-gated channel subfamily C member 1	-0.523
SLC23A2	solute carrier family 23 member 2	-0.528
SYNJ2	synaptojanin 2	-0.533
MTMR6	myotubularin related protein 6	-0.543
DGAT2	diacylglycerol O-acyltransferase 2	-0.547
glycyrrhetinic acid	--	-0.556
MAP2K1	mitogen-activated protein kinase kinase 1	-0.568
NQO1	NAD(P)H quinone dehydrogenase 1	-0.581
PFKFB2	6-phosphofructo-2-kinase/fructose-2,6-biphosphatase 2	-0.596
SORBS1	sorbin and SH3 domain containing 1	-0.596
CRY2	cryptochrome circadian regulator 2	-0.598
WWP1	WW domain containing E3 ubiquitin protein ligase 1	-0.603
OSTM1	osteopetrosis associated transmembrane protein 1	-0.607
uridine	--	-0.657
HK2	hexokinase 2	-0.658
PPP1R3A	protein phosphatase 1 regulatory subunit 3A	-0.678

GCLC	glutamate-cysteine ligase catalytic subunit	-0.688
PGK1	phosphoglycerate kinase 1	-0.749
CAT	catalase	-0.761
psychosine	--	-0.771
PRKAB2	protein kinase AMP-activated non-catalytic subunit beta 2	-0.779
HRASLS	HRAS like suppressor	-0.788
ITGAV	integrin subunit alpha V	-0.793
CNOT7	CCR4-NOT transcription complex subunit 7	-0.812
CHPF2	chondroitin polymerizing factor 2	-0.822
PLAU	plasminogen activator, urokinase	-0.838
2-hydroxyhexadecanoic acid	--	-0.872
ETNK1	ethanolamine kinase 1	-0.878
gliclazide	--	-0.878
NAGLU	N-acetyl-alpha-glucosaminidase	-0.9
PYGB	glycogen phosphorylase B	-0.904
1-14:0 lysophosphatidylcholine	--	-0.909
JAK2	Janus kinase 2	-0.911
ADP	--	-0.935
IRF5	interferon regulatory factor 5	-0.97
DPM1	dolichyl-phosphate mannosyltransferase subunit 1, catalytic	-1.063
NTRK3	neurotrophic receptor tyrosine kinase 3	-1.084
CCDC80	coiled-coil domain containing 80	-1.137
CEBPA	CCAAT enhancer binding protein alpha	-1.149
ESRRB	estrogen related receptor beta	-1.271
ATP2A2	ATPase sarcoplasmic/endoplasmic reticulum Ca2+ transporting 2	-1.275
CES1	carboxylesterase 1	-1.307
HSD11B1	hydroxysteroid 11-beta dehydrogenase 1	-1.423
INPP5J	inositol polyphosphate-5-phosphatase J	-1.441
POSTN	periostin	-1.67
TRARG1	trafficking regulator of GLUT4 (SLC2A4) 1	-1.923
GCK	glucokinase	-1.938
PER2	period circadian regulator 2	-2.426
PCK1	phosphoenolpyruvate carboxykinase 1	-3.131
NNAT	neuronatin	-4.149

**Table S2C. The non-canonical networks linked to musculoskeletal operations. Each table includes the gene symbol and gene name, and sorted based on their fold changes transformed to log scale base 2. (C) Lipid metabolism network was enriched by 49 upregulated and 98 downregulated biomolecules. The biomolecules included 119 genes and 28 metabolites.**		
Gene symbol	Entrez Gene Name	Expr Log Ratio
KLF5	Kruppel like factor 5	2.288
SOCS1	suppressor of cytokine signaling 1	2.023
FOS	Fos proto-oncogene, AP-1 transcription factor subunit	1.918
RRAD	RRAD, Ras related glycolysis inhibitor and calcium channel regulator	1.755
PNPLA3	patatin like phospholipase domain containing 3	1.74
NFIL3	nuclear factor, interleukin 3 regulated	1.663
CYP26B1	cytochrome P450 family 26 subfamily B member 1	1.578
CDKN1A	cyclin dependent kinase inhibitor 1A	1.558
UCN2	urocortin 2	1.556
ARNTL	aryl hydrocarbon receptor nuclear translocator like	1.531
IGF2BP2	insulin like growth factor 2 mRNA binding protein 2	1.504
TM6SF2	transmembrane 6 superfamily member 2	1.441

SEC14L2	SEC14 like lipid binding 2	1.374
NEU3	neuraminidase 3	1.32
SOCS3	suppressor of cytokine signaling 3	1.319
SLCO2A1	solute carrier organic anion transporter family member 2A1	1.268
CADM1	cell adhesion molecule 1	1.157
NR1D1	nuclear receptor subfamily 1 group D member 1	1.137
PLVAP	plasmalemma vesicle associated protein	1.128
ABCB1	ATP binding cassette subfamily B member 1	1.051
PPP1R3C	protein phosphatase 1 regulatory subunit 3C	0.943
ENHO	energy homeostasis associated	0.935
WNT4	Wnt family member 4	0.821
MVK	mevalonate kinase	0.787
ACACA	acetyl-CoA carboxylase alpha	0.704
SORT1	sortilin 1	0.691
oleoylethanolamide	--	0.665
DHRS3	dehydrogenase/reductase 3	0.656
DPEP1	dipeptidase 1	0.652
STAT5B	signal transducer and activator of transcription 5B	0.636
HS6ST1	heparan sulfate 6-O-sulfotransferase 1	0.609
VAC14	Vac14, PIKFYVE complex component	0.6
EBP	EBP, cholestenol delta-isomerase	0.587
SPHK2	sphingosine kinase 2	0.579
TECR	trans-2,3-enoyl-CoA reductase	0.547
RARA	retinoic acid receptor alpha	0.532
IL4R	interleukin 4 receptor	0.523
APOD	apolipoprotein D	0.516
dioleoylphosphatidylglycerol	--	0.497
MIF	macrophage migration inhibitory factor	0.494
SMAD3	SMAD family member 3	0.493
GCDH	glutaryl-CoA dehydrogenase	0.488

BSCL2	BSCL2, seipin lipid droplet biogenesis associated	0.487
NFATC2	nuclear factor of activated T cells 2	0.458
GRB10	growth factor receptor bound protein 10	0.441
SIK3	SIK family kinase 3	0.43
TGM2	transglutaminase 2	0.393
mono-(2-ethylhexyl)phthalate	--	0.249
dibutyl phthalate	--	0.249
beta-alanine	--	-0.288
urethane	--	-0.288
hyperforin	--	-0.302
ATP	--	-0.347
HSPA5	heat shock protein family A (Hsp70) member 5	-0.359
acetic acid	--	-0.388
glycolaldehyde	--	-0.388
MAPK1	mitogen-activated protein kinase 1	-0.388
RAB14	RAB14, member RAS oncogene family	-0.395
SH3GLB1	SH3 domain containing GRB2 like, endophilin B1	-0.41
SRSF2	serine and arginine rich splicing factor 2	-0.411
RTN4	reticulon 4	-0.415
pyridoxamine	--	-0.424
acetaminophen	--	-0.424
PDHB	pyruvate dehydrogenase E1 beta subunit	-0.424
CHPT1	choline phosphotransferase 1	-0.44
carnosine	--	-0.451
CXCL14	C-X-C motif chemokine ligand 14	-0.454
HADH	hydroxyacyl-CoA dehydrogenase	-0.476
P2RY2	purinergic receptor P2Y2	-0.477
DHRS11	dehydrogenase/reductase 11	-0.486
gentian violet	--	-0.489
RHOQ	ras homolog family member Q	-0.493
ATP8A1	ATPase phospholipid transporting 8A1	-0.497

ST3GAL5	ST3 beta-galactoside alpha-2,3-sialyltransferase 5	-0.497
PTGES2	prostaglandin E synthase 2	-0.499
ACAT1	acetyl-CoA acetyltransferase 1	-0.504
CPE	carboxypeptidase E	-0.507
niacinamide	--	-0.514
PTPN11	protein tyrosine phosphatase, non-receptor type 11	-0.515
TEF	TEF, PAR bZIP transcription factor	-0.516
EXTL1	exostosin like glycosyltransferase 1	-0.518
SLC23A2	solute carrier family 23 member 2	-0.528
SYNJ2	synaptojanin 2	-0.533
MTMR6	myotubularin related protein 6	-0.543
CCNY	cyclin Y	-0.547
DGAT2	diacylglycerol O-acyltransferase 2	-0.547
LGR4	leucine rich repeat containing G protein-coupled receptor 4	-0.548
STX12	syntaxin 12	-0.549
glycyrrhetinic acid	--	-0.556
adenine	--	-0.568
MAP2K1	mitogen-activated protein kinase kinase 1	-0.568
ABCD3	ATP binding cassette subfamily D member 3	-0.571
PHYH	phytanoyl-CoA 2-hydroxylase	-0.571
NQO1	NAD(P)H quinone dehydrogenase 1	-0.581
CANX	calnexin	-0.582
BAG3	BCL2 associated athanogene 3	-0.586
PFKFB2	6-phosphofructo-2-kinase/fructose-2,6-biphosphatase 2	-0.596
SORBS1	sorbin and SH3 domain containing 1	-0.596
CRY2	cryptochrome circadian regulator 2	-0.598

SDHA	succinate dehydrogenase complex flavoprotein subunit A	-0.604
PLAA	phospholipase A2 activating protein	-0.625
SOX17	SRY-box 17	-0.627
SSPN	sarcospan	-0.629
FN1	fibronectin 1	-0.65
uridine	--	-0.657
DLD	dihydrolipoamide dehydrogenase	-0.675
SUCLA2	succinate-CoA ligase ADP-forming beta subunit	-0.702
L-alpha-lysophosphatidylcholine, stearoyl	--	-0.72
TMEM30A	transmembrane protein 30A	-0.724
CBR4	carbonyl reductase 4	-0.739
DLAT	dihydrolipoamide S-acetyltransferase	-0.758
sphingosyl beta-glucoside	--	-0.771
psychosine	--	-0.771
PRKAB2	protein kinase AMP-activated non-catalytic subunit beta 2	-0.779
HRASLS	HRAS like suppressor	-0.788
ITGAV	integrin subunit alpha V	-0.793
LPGAT1	lysophosphatidylglycerol acyltransferase 1	-0.81
QKI	QKI, KH domain containing RNA binding	-0.821
saquinavir	--	-0.828
PLAU	plasminogen activator, urokinase	-0.838
UBR1	ubiquitin protein ligase E3 component n-recognin 1	-0.855
ginsenoside Rb1	--	-0.861
2-hydroxyhexadecanoic acid	--	-0.872
ETNK1	ethanolamine kinase 1	-0.878
NAGLU	N-acetyl-alpha-glucosaminidase	-0.9
JAK2	Janus kinase 2	-0.911
HLF	HLF, PAR bZIP transcription factor	-0.915
ADP	--	-0.935

ADH1C	alcohol dehydrogenase 1C (class I), gamma polypeptide	-0.944
FGF9	fibroblast growth factor 9	-0.995
DPM1	dolichyl-phosphate mannosyltransferase subunit 1, catalytic	-1.063
CCDC80	coiled-coil domain containing 80	-1.137
CEBPA	CCAAT enhancer binding protein alpha	-1.149
cholestane-3beta,5alpha,6beta-triol	--	-1.166
IL15	interleukin 15	-1.176
FGFR3	fibroblast growth factor receptor 3	-1.267
SLC16A1	solute carrier family 16 member 1	-1.269
ATP2A2	ATPase sarcoplasmic/endoplasmic reticulum Ca2+ transporting 2	-1.275
CES1	carboxylesterase 1	-1.307
NUDT7	nudix hydrolase 7	-1.419
HSD11B1	hydroxysteroid 11-beta dehydrogenase 1	-1.423
INPP5J	inositol polyphosphate-5-phosphatase J	-1.441
methylprednisolone	--	-1.542
GCK	glucokinase	-1.938
PER2	period circadian regulator 2	-2.426
tetradecanal	--	-2.452
PCK1	phosphoenolpyruvate carboxykinase 1	-3.131

Table S2D. The non-canonical networks linked to musculoskeletal operations. Each table includes the gene symbol and gene name, and sorted based on their fold changes transformed to log scale base 2. (D) Muscle function network was enriched by 51 upregulated and 60 downregulated biomolecules. The biomolecules included 108 genes and 3 metabolites. The molecules linked to calcium ion (Ca+2) channel are highlighted.

Gene symbol	Entrez Gene Name	Expr Log Ratio
KLF5	Kruppel like factor 5	2.288
SOCS1	suppressor of cytokine signaling 1	2.023
FOS	Fos proto-oncogene, AP-1 transcription factor subunit	1.918
RRAD	RRAD, Ras related glycolysis inhibitor and calcium channel regulator	1.755
CDKN1A	cyclin dependent kinase inhibitor 1A	1.558
UCN2	urocortin 2	1.556
ARNTL	aryl hydrocarbon receptor nuclear translocator like	1.531
NEU3	neuraminidase 3	1.32
SOCS3	suppressor of cytokine signaling 3	1.319
FST	follistatin	1.238
MYBPH	myosin binding protein H	1.168
TP63	tumor protein p63	1.086
PEG3	paternally expressed 3	1.049
ACVR2B	activin A receptor type 2B	1.019
PIM1	Pim-1 proto-oncogene, serine/threonine kinase	1.003
WNT4	Wnt family member 4	0.821
CNP	2',3'-cyclic nucleotide 3' phosphodiesterase	0.772
CHRNB1	cholinergic receptor nicotinic beta 1 subunit	0.741
FGFR1	fibroblast growth factor receptor 1	0.725
SORT1	sortilin 1	0.691
STAT5B	signal transducer and activator of transcription 5B	0.636
Foxp1	forkhead box P1	0.626
SLC8A1	solute carrier family 8 member A1	0.626
PDE2A	phosphodiesterase 2A	0.622
ZFHX3	zinc finger homeobox 3	0.607

TRIM63	tripartite motif containing 63	0.589
SLC9A1	solute carrier family 9 member A1	0.585
APOBEC2	apolipoprotein B mRNA editing enzyme catalytic subunit 2	0.561
VASP	vasodilator stimulated phosphoprotein	0.538
RARA	retinoic acid receptor alpha	0.532
TRIO	trio Rho guanine nucleotide exchange factor	0.527
IL4R	interleukin 4 receptor	0.523
FMOD	fibromodulin	0.519
FXYD1	FXYD domain containing ion transport regulator 1	0.518
CDH15	cadherin 15	0.512
MYO18B	myosin XVIIIB	0.504
SMAD3	SMAD family member 3	0.493
PPP1R1A	protein phosphatase 1 regulatory inhibitor subunit 1A	0.492
YAP1	Yes associated protein 1	0.492
MYL12A	myosin light chain 12A	0.473
NFATC2	nuclear factor of activated T cells 2	0.458
GRB10	growth factor receptor bound protein 10	0.441
LMO7	LIM domain 7	0.439
PXN	paxillin	0.409
TNNI2	troponin I2, fast skeletal type	0.409
ALDOA	aldolase, fructose-bisphosphate A	0.406
TGM2	transglutaminase 2	0.393
HSF1	heat shock transcription factor 1	0.391
SPEG	SPEG complex locus	0.364
IL6ST	interleukin 6 signal transducer	0.359
MYH4	myosin heavy chain 4	0.311
ATP	--	-0.347
MAPK1	mitogen-activated protein kinase 1	-0.388
UBR2	ubiquitin protein ligase E3 component n-recognin 2	-0.428
SPAG9	sperm associated antigen 9	-0.437
CXCL14	C-X-C motif chemokine ligand 14	-0.454
TARBP2	TARBP2, RISC loading complex RNA binding subunit	-0.456
CFL2	cofilin 2	-0.481

PLEKHO1	pleckstrin homology domain containing O1	-0.508
MYOT	myotilin	-0.51
niacinamide	--	-0.514
PTPN11	protein tyrosine phosphatase, non-receptor type 11	-0.515
UCHL3	ubiquitin C-terminal hydrolase L3	-0.557
RB1CC1	RB1 inducible coiled-coil 1	-0.564
MAP2K1	mitogen-activated protein kinase kinase 1	-0.568
EIF2AK4	eukaryotic translation initiation factor 2 alpha kinase 4	-0.577
SORBS1	sorbin and SH3 domain containing 1	-0.596
ACTN2	actinin alpha 2	-0.602
ADK	adenosine kinase	-0.611
RCAN2	regulator of calcineurin 2	-0.614
JARID2	jumonji and AT-rich interaction domain containing 2	-0.622
SSPN	sarcospan	-0.629
GPCPD1	glycerophosphocholine phosphodiesterase 1	-0.63
CRK	CRK proto-oncogene, adaptor protein	-0.645
FN1	fibronectin 1	-0.65
LBX1	ladybird homeobox 1	-0.653
CKMT2	creatine kinase, mitochondrial 2	-0.663
MYL4	myosin light chain 4	-0.669
MYH1	myosin heavy chain 1	-0.672
BACH1	BTB domain and CNC homolog 1	-0.673
FHOD3	formin homology 2 domain containing 3	-0.752
CAT	catalase	-0.761
PRKAB2	protein kinase AMP-activated non-catalytic subunit beta 2	-0.779
ITGAV	integrin subunit alpha V	-0.793
MYBPC1	myosin binding protein C, slow type	-0.806
LMOD2	leiomodin 2	-0.814
GJA1	gap junction protein alpha 1	-0.821
QKI	QKI, KH domain containing RNA binding	-0.821
PLAU	plasminogen activator, urokinase	-0.838

UBR1	ubiquitin protein ligase E3 component n-recognin 1	-0.855
ATP1B1	ATPase Na+/K+ transporting subunit beta 1	-0.863
MB	myoglobin	-0.904
JAK2	Janus kinase 2	-0.911
HLF	HLF, PAR bZIP transcription factor	-0.915
ADP	--	-0.935
MYOD1	myogenic differentiation 1	-1.01
CD55	CD55 molecule (Cromer blood group)	-1.045
TPM3	tropomyosin 3	-1.095
CEBPA	CCAAT enhancer binding protein alpha	-1.149
FHL1	four and a half LIM domains 1	-1.193
ATP2A2	ATPase sarcoplasmic/endoplasmic reticulum Ca2+ transporting 2	-1.275
HSD11B1	hydroxysteroid 11-beta dehydrogenase 1	-1.423
MYH2	myosin heavy chain 2	-1.473
CSRP3	cysteine and glycine rich protein 3	-2.214
MYOZ2	myozenin 2	-2.245
ANKRD2	ankyrin repeat domain 2	-2.484
MYL2	myosin light chain 2	-3.344
TNNT1	troponin T1, slow skeletal type	-3.629
TNNC1	troponin C1, slow skeletal and cardiac type	-3.922
TNNI1	troponin I1, slow skeletal type	-4.478
MYH7	myosin heavy chain 7	-5.235

Table S2E. The non-canonical networks linked to musculoskeletal operations. Each table includes the gene symbol and gene name, and sorted based on their fold changes transformed to log scale base 2. (E) Inflammation network was enriched by 64 upregulated and 89 downregulated biomolecules. The biomolecules included 114 genes and 39 metabolites.

Gene symbol	Entrez Gene Name	Expr Log Ratio
FOSB	FosB proto-oncogene, AP-1 transcription factor subunit	2.351
SOCS1	suppressor of cytokine signaling 1	2.023
FOS	Fos proto-oncogene, AP-1 transcription factor subunit	1.918
NFIL3	nuclear factor, interleukin 3 regulated	1.663
CYP26B1	cytochrome P450 family 26 subfamily B member 1	1.578
CDKN1A	cyclin dependent kinase inhibitor 1A	1.558
SOCS3	suppressor of cytokine signaling 3	1.319
NR1D1	nuclear receptor subfamily 1 group D member 1	1.137
TP63	tumor protein p63	1.086
PTPN22	protein tyrosine phosphatase, non-receptor type 22	1.067
CEBPD	CCAAT enhancer binding protein delta	1.054
ABCB1	ATP binding cassette subfamily B member 1	1.051
PPP1R15A	protein phosphatase 1 regulatory subunit 15A	0.883
MVK	mevalonate kinase	0.787
CNP	2',3'-cyclic nucleotide 3' phosphodiesterase	0.772
EGLN3	egl-9 family hypoxia inducible factor 3	0.727
FGFR1	fibroblast growth factor receptor 1	0.725
MMP15	matrix metallopeptidase 15	0.711
oleoylethanolamide	--	0.665
STAT5B	signal transducer and activator of transcription 5B	0.636
ALPK2	alpha kinase 2	0.627
PDE2A	phosphodiesterase 2A	0.622
PFKFB3	6-phosphofructo-2-kinase/fructose-2,6-biphosphatase 3	0.612
SLC9A1	solute carrier family 9 member A1	0.585
SPHK2	sphingosine kinase 2	0.579
SDC4	syndecan 4	0.573
ERRFI1	ERBB receptor feedback inhibitor 1	0.533
TRIO	trio Rho guanine nucleotide exchange factor	0.527
IL4R	interleukin 4 receptor	0.523
FMOD	fibromodulin	0.519
MFGE8	milk fat globule-EGF factor 8 protein	0.518

RPL11	ribosomal protein L11	0.506
dioctyl adipate	--	0.501
RPL13A	ribosomal protein L13a	0.497
MIF	macrophage migration inhibitory factor	0.494
SMAD3	SMAD family member 3	0.493
YAP1	Yes associated protein 1	0.492
BSG	basigin (Ok blood group)	0.49
KARS	lysyl-tRNA synthetase	0.484
DDT	D-dopachrome tautomerase	0.473
MYL12A	myosin light chain 12A	0.473
DYM	dymeclin	0.472
BCL6	B cell CLL/lymphoma 6	0.464
NFATC2	nuclear factor of activated T cells 2	0.458
KSR1	kinase suppressor of ras 1	0.453
CACNG1	calcium voltage-gated channel auxiliary subunit gamma 1	0.447
RPL18A	ribosomal protein L18a	0.445
GRB10	growth factor receptor bound protein 10	0.441
RHOB	ras homolog family member B	0.441
EIF2AK1	eukaryotic translation initiation factor 2 alpha kinase 1	0.438
MYO18A	myosin XVIIIA	0.427
AMPD1	adenosine monophosphate deaminase 1	0.413
PXN	paxillin	0.409
PPP1R7	protein phosphatase 1 regulatory subunit 7	0.407
ALDOA	aldolase, fructose-bisphosphate A	0.406
RPSA	ribosomal protein SA	0.394
TGM2	transglutaminase 2	0.393
HSF1	heat shock transcription factor 1	0.391
RPL31	ribosomal protein L31	0.375
RPS18	ribosomal protein S18	0.367
TRPM4	transient receptor potential cation channel subfamily M member 4	0.366
MAPT	microtubule associated protein tau	0.364
IL6ST	interleukin 6 signal transducer	0.359
mono-(2-ethylhexyl)phthalate	--	0.249
L-alanine	--	-0.288
pyruvaldehyde	--	-0.288
malondialdehyde	--	-0.288
urethane	--	-0.288
alanine	--	-0.288
hyperforin	--	-0.302
ATP	--	-0.347
HSPA5	heat shock protein family A (Hsp70) member 5	-0.359
acetic acid	--	-0.388

MCL1	MCL1, BCL2 family apoptosis regulator	-0.412
acetaminophen	--	-0.424
carnosine	--	-0.451
CXCL14	C-X-C motif chemokine ligand 14	-0.454
SERINC3	serine incorporator 3	-0.49
ATP8A1	ATPase phospholipid transporting 8A1	-0.497
CPE	carboxypeptidase E	-0.507
ADSS	adenylosuccinate synthase	-0.51
niacinamide	--	-0.514
PTPN11	protein tyrosine phosphatase, non-receptor type 11	-0.515
PDE7A	phosphodiesterase 7A	-0.55
1-oleoyl-lysophosphatidylethanolamine	--	-0.556
glycyrrhetinic acid	--	-0.556
RB1CC1	RB1 inducible coiled-coil 1	-0.564
adenine	--	-0.568
MAP2K1	mitogen-activated protein kinase kinase 1	-0.568
ITFG1	integrin alpha FG-GAP repeat containing 1	-0.573
CDK19	cyclin dependent kinase 19	-0.575
HLA-DRB5	major histocompatibility complex, class II, DR beta 5	-0.576
EIF2AK4	eukaryotic translation initiation factor 2 alpha kinase 4	-0.577
PCM1	pericentriolar material 1	-0.577
NQO1	NAD(P)H quinone dehydrogenase 1	-0.581
CRY2	cryptochrome circadian regulator 2	-0.598
NR1D2	nuclear receptor subfamily 1 group D member 2	-0.6
2-16:0 lysophosphatidylethanolamine	--	-0.607
1-palmitoyl-2-hydroxy-sn-glycero-3-phosphoethanolamine	--	-0.607
OSTM1	osteopetrosis associated transmembrane protein 1	-0.607
HLA-DQA1	major histocompatibility complex, class II, DQ alpha 1	-0.611
PLAA	phospholipase A2 activating protein	-0.625
1-(1Z-hexadecenyl)-sn-glycero-3-phosphoethanolamine	--	-0.626
MAP3K3	mitogen-activated protein kinase kinase kinase 3	-0.63
NOTCH4	notch 4	-0.64

FN1	fibronectin 1	-0.65
uridine	--	-0.657
pseudouridine	--	-0.657
TFRC	transferrin receptor	-0.672
NDUFC2	NADH:ubiquinone oxidoreductase subunit C2	-0.701
L-palmitoylcarnitine	--	-0.704
L-alpha-lysophosphatidylcholine, stearoyl	--	-0.72
1-20:4(5Z,8Z,11Z,14Z) lysophosphatidylcholine	--	-0.72
1-18:2(9Z,12Z) lysophosphatidylcholine	--	-0.727
PGK1	phosphoglycerate kinase 1	-0.749
1-oleoyl lysophosphatidylcholine	--	-0.751
1-heptadecanoyl-2-hydroxy-sn-glycero-3-phosphocholine	--	-0.752
WIPF1	WAS/WASL interacting protein family member 1	-0.76
psychosine	--	-0.771
ADAMTS5	ADAM metallopeptidase with thrombospondin type 1 motif 5	-0.775
LOXL2	lysyl oxidase like 2	-0.78
5'-methylthioadenosine	--	-0.782
ITGAV	integrin subunit alpha V	-0.793
GJA1	gap junction protein alpha 1	-0.821
QKI	QKI, KH domain containing RNA binding	-0.821
ADAMTS1	ADAM metallopeptidase with thrombospondin type 1 motif 1	-0.832
PLAU	plasminogen activator, urokinase	-0.838
PHTF1	putative homeodomain transcription factor 1	-0.848
ginsenoside Rb1	--	-0.861
ATP1B1	ATPase Na+/K+ transporting subunit beta 1	-0.863
N-acetyl-L-phenylalanine	--	-0.87
2-hydroxyhexadecanoic acid	--	-0.872
inosine	--	-0.876
NAGLU	N-acetyl-alpha-glucosaminidase	-0.9
1-14:0 lysophosphatidylcholine	--	-0.909
JAK2	Janus kinase 2	-0.911
ADP	--	-0.935

ADAMTS14	ADAM metallopeptidase with thrombospondin type 1 motif 14	-0.943
IRF5	interferon regulatory factor 5	-0.97
MST1R	macrophage stimulating 1 receptor	-0.982
oleoylcarnitine	--	-0.994
Retnla	resistin like alpha	-1.002
SLC1A5	solute carrier family 1 member 5	-1.013
CEBPA	CCAAT enhancer binding protein alpha	-1.149
IL15	interleukin 15	-1.176
PDE7B	phosphodiesterase 7B	-1.176
adenine-riboflavin dinucleotide	--	-1.184
SLC7A2	solute carrier family 7 member 2	-1.222
FGFR3	fibroblast growth factor receptor 3	-1.267
CES1	carboxylesterase 1	-1.307
methylprednisolone	--	-1.542
POSTN	periostin	-1.67
TNNC1	troponin C1, slow skeletal and cardiac type	-3.922

**Table S2F. The non-canonical networks linked to musculoskeletal operations. Each table includes the gene symbol and gene name, and sorted based on their fold changes transformed to log scale base 2. (F) Mitochondrial dysfunction network was enriched by 18 upregulated and 40 downregulated biomolecules. The biomolecules included 42 genes and 16 metabolites.**		
Gene symbol	Entrez Gene Name	Expr Log Ratio
SLCO2A1	solute carrier organic anion transporter family member 2A1	1.268
TP63	tumor protein p63	1.086
NT5C2	5'-nucleotidase, cytosolic II	0.763
ACACA	acetyl-CoA carboxylase alpha	0.704
oleoylethanolamide	--	0.665
COX6A2	cytochrome c oxidase subunit 6A2	0.572
ENTPD6	ectonucleoside triphosphate diphosphohydrolase 6 (putative)	0.572
VASP	vasodilator stimulated phosphoprotein	0.538
MIF	macrophage migration inhibitory factor	0.494
GCDH	glutaryl-CoA dehydrogenase	0.488
FIS1	fission, mitochondrial 1	0.452
EIF2AK1	eukaryotic translation initiation factor 2 alpha kinase 1	0.438

HSD17B10	hydroxysteroid 17-beta dehydrogenase 10	0.429
MYO18A	myosin XVIIIA	0.427
ALDOA	aldolase, fructose-bisphosphate A	0.406
TGM2	transglutaminase 2	0.393
lisuride	--	0.379
dibutyl phthalate	--	0.249
pyruvaldehyde	--	-0.288
malondialdehyde	--	-0.288
alanine	--	-0.288
hyperforin	--	-0.302
ATP	--	-0.347
HSPA5	heat shock protein family A (Hsp70) member 5	-0.359
NDUFS1	NADH:ubiquinone oxidoreductase core subunit S1	-0.375
MAPK1	mitogen-activated protein kinase 1	-0.388
COX5A	cytochrome c oxidase subunit 5A	-0.406
CCT4	chaperonin containing TCP1 subunit 4	-0.41
acetaminophen	--	-0.424
NDUFB6	NADH:ubiquinone oxidoreductase subunit B6	-0.429
UQCRFS1	ubiquinol-cytochrome c reductase, Rieske iron-sulfur polypeptide 1	-0.441
HADH	hydroxyacyl-CoA dehydrogenase	-0.476
ATP5PB	ATP synthase peripheral stalk-membrane subunit b	-0.519
NDUFAF1	NADH:ubiquinone oxidoreductase complex assembly factor 1	-0.544
DGAT2	diacylglycerol O-acyltransferase 2	-0.547
glycyrrhetinic acid	--	-0.556
UCHL3	ubiquitin C-terminal hydrolase L3	-0.557

SDHD	succinate dehydrogenase complex subunit D	-0.565
adenine	--	-0.568
ABCD3	ATP binding cassette subfamily D member 3	-0.571
PHYH	phytanoyl-CoA 2-hydroxylase	-0.571
NQO1	NAD(P)H quinone dehydrogenase 1	-0.581
SDHA	succinate dehydrogenase complex flavoprotein subunit A	-0.604
HADHB	hydroxyacyl-CoA dehydrogenase trifunctional multienzyme complex subunit beta	-0.663
ATP5S	ATP synthase, H+ transporting, mitochondrial Fo complex subunit s (factor B)	-0.702
L-palmitoylcarnitine	--	-0.704
AOX1	aldehyde oxidase 1	-0.733
CAT	catalase	-0.761
psychosine	--	-0.771
ATP5MC3	ATP synthase membrane subunit c locus 3	-0.778
inosine	--	-0.876
MB	myoglobin	-0.904
ADP	--	-0.935
ADH1C	alcohol dehydrogenase 1C (class I), gamma polypeptide	-0.944
IL15	interleukin 15	-1.176
SLC16A1	solute carrier family 16 member 1	-1.269
HSD11B1	hydroxysteroid 11-beta dehydrogenase 1	-1.423
methylprednisolone	--	-1.542

Table S3. The list of genes associated with the upstream regulators of interest, namely SOCS1, STAT5B, SMAD3, HSF1, mono-(2-ethylhexyl) phthalate and PLAU. For individual upstream regulator, the gene symbols were sorted in descending order.											
**Upstream regulator: SOCS1**		**Upstream regulator: STAT5B**		**Upstream regulator: SMAD3**		**Upstream regulator: HSF1**		**Upstream regulator: mono-(2-ethylhexyl) phthalate**		**Upstream regulator: PLAU**	
**Gene symbol**	**Expr Log Ratio**	**Gene symbol**	**Expr Log Ratio**	**Gene symbol**	**Expr Log Ratio**	**Gene symbol**	**Expr Log Ratio**	**Gene symbol**	**Expr Log Ratio**	**Gene symbol**	**Expr Log Ratio**
SOCS1	2.02	FAM89A	2.29	FOS	1.92	RRAD	1.76	FOS	1.92	HSPA5	-0.36
FOS	1.92	SOCS1	2.02	CDKN1A	1.56	FBLN1	1.70	DDIT4	1.41	FN1	-0.65
CISH	1.89	FOS	1.92	FST	1.24	ABCB1	1.05	BSCL2	0.49	ITGAV	-0.79
CDKN1A	1.56	CISH	1.89	PMEPA1	0.53	UBC	0.89	NDUFS1	-0.38	GJA1	-0.82
SOCS3	1.32	FBLN1	1.70	JUND	0.52	SPHK2	0.58	PDHB	-0.42	PLAU	-0.84
IL4R	0.52	CYP26B1	1.58	SMAD3	0.49	FMOD	0.52	UQCRFS1	-0.44	Retnla	-1.00
TGM2	0.39	CDKN1A	1.56	RHOB	0.44	PTOV1	0.48	HADH	-0.48		
JAK2	-0.91	SOCS3	1.32	GLS	-0.51	NFATC2	0.46	ATP5PB	-0.52		
Retnla	-1.00	ABCB1	1.05	FN1	-0.65	HSF1	0.39	DGAT2	-0.55		
IL15	-1.18	PIM1	1.00	stearoyl	-0.72	MAPT	0.36	SDHD	-0.57		
		PPP1R3C	0.94	1-oleoyl lysophosphatidylcholine	-0.75	CCT4	-0.41	SDHA	-0.60		
		STAT5B	0.64	MYOD1	-1.01	MCL1	-0.41	HK2	-0.66		
		COX6A2	0.57	TPM3	-1.10	EIF4A2	-0.57	PGK1	-0.75		
		BCL6	0.46	IL15	-1.18	BAG3	-0.59	CEBPA	-1.15		
		KSR1	0.45			JARID2	-0.62	GCK	-1.94		
		MCL1	-0.41			PGK1	-0.75	PCK1	-3.13		
		DGAT2	-0.55			ZFAND2A	-0.75				
		NQO1	-0.58			DNAJA1	-1.01				

		SLC47A1	-0.64			CCT6A	-1.21				
		CKMT2	-0.66								
		MYL4	-0.67								
		MYH1	-0.67								
		MB	-0.90								
		Retnla	-1.00								
		TPM3	-1.10								
		ATP2A2	-1.28								
		NUDT7	-1.42								
		GCK	-1.94								
		CSRP3	-2.21								
		MYL2	-3.34								
		TNNT1	-3.63								
		TNNC1	-3.92								
		TNNI1	-4.48								
		MYH7	-5.24								
